# Supplementary material for: Targeted pandemic containment through identifying local contact network bottlenecks
Source: PLoS Comput Biol. 2021 Aug 30;17(8):e1009351. doi: 10.1371/journal.pcbi.1009351 (PMC8432902; doi:10.1371/journal.pcbi.1009351)
Supplement: S1 Text — (PDF) [file pcbi.1009351.s001.pdf]

S1 Text. CF betweenness is a special case of ℓ_2-flow betweenness

Fix $\Delta = \mathbf{1}_s$ and $T = \mathbf{1}_t$ for some $s, t \in V$. Let B denote the signed arc-node incidence matrix using an arbitrary orientation of the edges, i.e., the row of edge (u, v) has two non-zero entries, -1 at column u and 1 at column v. Then the optimization problem (see Eq (1) in the main text) giving rise to ℓ_2-flow betweenness can be more succinctly written as

$$\text{minimize } \|f\|_2^2 \quad \text{subject to} \quad B^T f + \mathbf{1}_s \leq \mathbf{1}_t,$$

or equivalently,

$$\text{minimize } \|f\|_2^2 \quad \text{subject to} \quad B^T f + \mathbf{1}_s = \mathbf{1}_t, \tag{1}$$

where $\|\cdot\|_2$ denotes the ℓ_2-norm. The equality constraint in the second formulation is due to the fact $\|\mathbf{1}_s\|_1 = \|\mathbf{1}_t\|_1$, where $\|\cdot\|_1$ denotes the ℓ_1-norm. Let f_{st} denote the optimal solution of Problem (1). Then by the optimality condition of Problem (1), f_{st} satisfies, for some $y \in \mathbb{R}^{|V|}$,

$$f_{st} + By = 0, \tag{2}$$

$$-B^T f_{st} = \mathbf{1}_s - \mathbf{1}_t. \tag{3}$$

Pre-multiply both sides of Eq (2) by B^T, and substitute Eq (3), we have

$$Ly = B^T By = -B^T f_{st} = \mathbf{1}_s - \mathbf{1}_t, \tag{4}$$

where L is the Laplacian matrix of G. Notice that Eq (4) is exactly the Laplacian linear system that defines the absolute potentials y of a unique st-current τ_{st} (e.g., see Lemma 3 in [1]), where $\tau(e) = y(u) - y(v)$ for $e = (u, v) \in E$. But then according to Eq (2) we have that $f_{st} = -By$, which implies

$$|f_{st}(e)| = |y(u) - y(v)|, \quad \text{for } e = (u, v) \in E.$$

Therefore $|f_{st}(e)| = |\tau_{st}(e)|$ for all e. Moreover, if $s = t$, then one simply has that $f_{st}(e) = 0$ for all e. Let $n = |V|$. It follows that

$$\mathbb{E}_{\Delta \sim \mathcal{U}_V, T \sim \mathcal{U}_V}[|f^*_{\Delta,T}(e)|] = \frac{1}{n^2} \sum_{s,t \in V} |f_{st}(e)| = \frac{1}{n^2} \sum_{s,t \in V, s \neq t} |f_{st}(e)| = \frac{1}{n^2} \sum_{s,t \in V, s \neq t} |\tau_{st}(e)|,$$

which is exactly the CF betweenness [1] normalized by $1/n^2$.

References

1. Brandes U, Fleischer D. Centrality Measures Based on Current Flow. In: Diekert V, Durand B, editors. STACS 2005. Berlin, Heidelberg: Springer Berlin Heidelberg; 2005. p. 533–544.
