# Supplementary material for: Targeted pandemic containment through identifying local contact network bottlenecks
Source: PLoS Comput Biol. 2021 Aug 30;17(8):e1009351. doi: 10.1371/journal.pcbi.1009351 (PMC8432902; doi:10.1371/journal.pcbi.1009351)
Supplement: S2 Text — (PDF) [file pcbi.1009351.s002.pdf]

S2 Text. Experiment on LFR synthetic graph

Lancichinetti–Fortunato–Radicchi (LFR) model [1] is a random graph generative model that produces synthetic graphs with a priori known communities. It is a widely used benchmark for testing community detection and local graph clustering algorithms. We carry out additional experiment on an LFR synthetic graph to illustrate how the local clustering bias of LF leads to more effective targeted interventions. We generated an LFR synthetic graph on 10,000 nodes, where we consider each node as an individual person. Node degrees and community sizes in the LFR model are distributed according to the power law distribution, we set the exponent for the degree sequence to 2 and the exponent for the community size distribution to 1.5. Moreover, we set the average node degree to 5, the maximum node degree to 50, the minimum community size to 20 and the maximum community size to 50. This roughly corresponds to the setting where small groups of individuals (families, friends, colleagues) constitute closely connected communities. We set the mixing parameter $\mu = 0.1$. The resulting graph has 38,313 edges and 323 known communities. Out of the 38,313 edges, 4,363 edges are the "cut" edges that connect one community to another. Intuitively, targeting these edges should be more effective at reducing epidemic spread than targeting edges that connect two nodes within the same community. The significance of targeting the "cut" edges on intervention effectiveness is evidently supported by our results shown in S8 Fig.

S8 Fig contains two plots that demonstrate, respectively, the final epidemic sizes using different interventions and the percentage of "cut" edges targeted by different betweenness measures as we vary the percentage of targeted edges. Observe the close relationship between the number of targeted "cut" edges and the final epidemic sizes: The more "cut" edges targeted, the more effective the intervention strategy is. For example, at every intervention coverage level, LF with $\lambda \in \{1/2, 1/10\}$ identifies and targets the highest number of "cut" edges, and this leads to the most effective interventions. At 13% intervention coverage level, CF targets more "cut" edges than SP, making CF more effective at reducing the final size than SP; similarly, at 15% intervention coverage level, CF surpasses LF ($\lambda = 1/50$) in the number of targeted "cut" edges (as shown in the zoomed window), and hence CF becomes more effective. Overall, because LF tends to detect edges that are bottleneck connections between or within small communities, it is most effective at identifying the "cut" edges on this synthetic LFR graph, which in turn helps produce the most effective intervention strategies.

References

1. Lancichinetti A, Fortunato S, Radicchi F. Benchmark graphs for testing community detection algorithms. Physical Review E. 2008;78(4). doi:10.1103/physreve.78.046110.
