# Supplementary figures and images for: Targeted pandemic containment through identifying local contact network bottlenecks
Source: PLoS Comput Biol. 2021 Aug 30;17(8):e1009351. doi: 10.1371/journal.pcbi.1009351 (PMC8432902; doi:10.1371/journal.pcbi.1009351)

**A**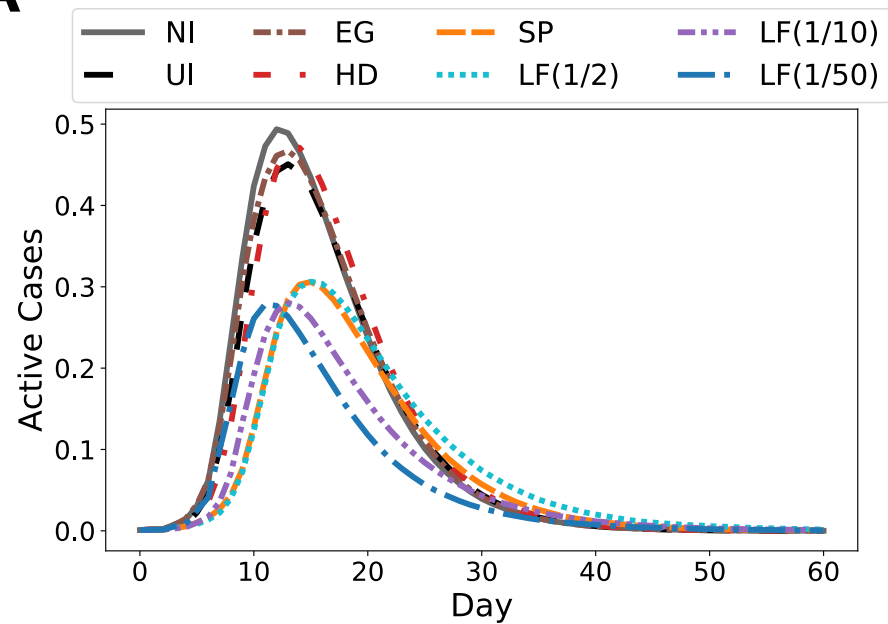**B**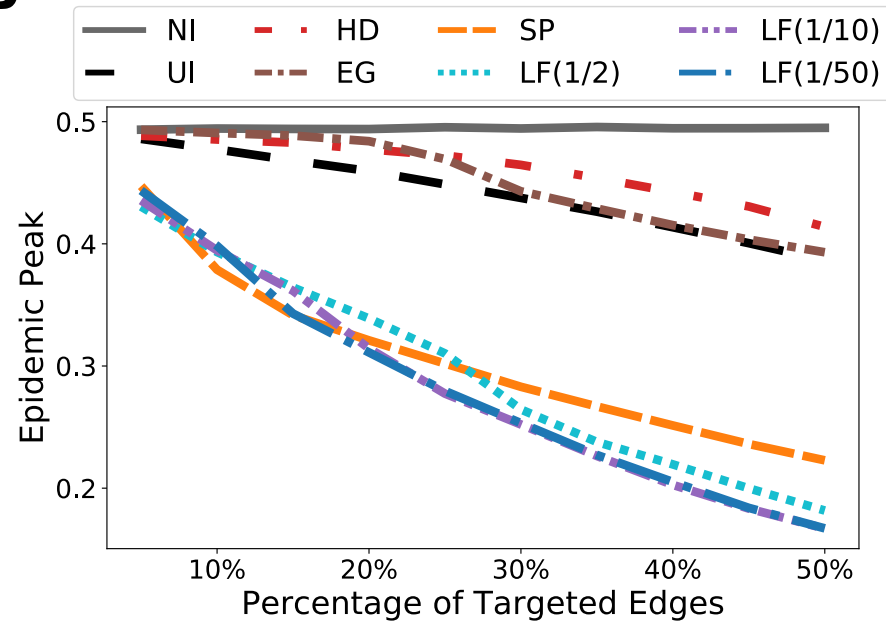**C**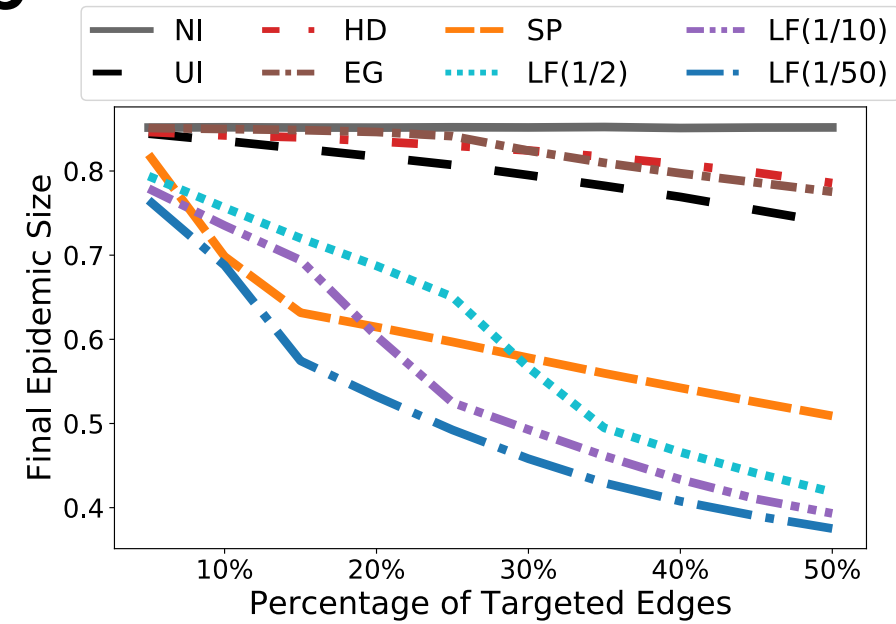**D**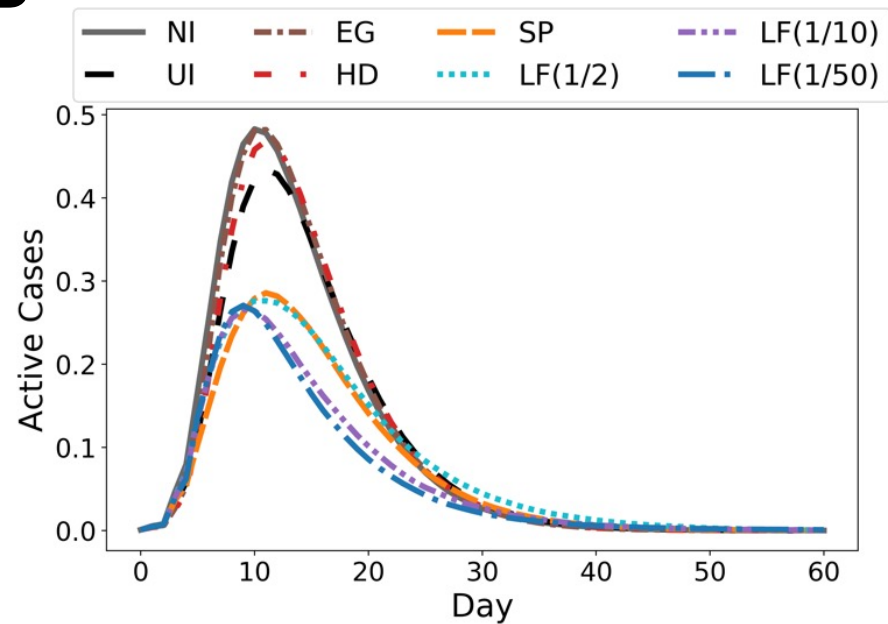**E**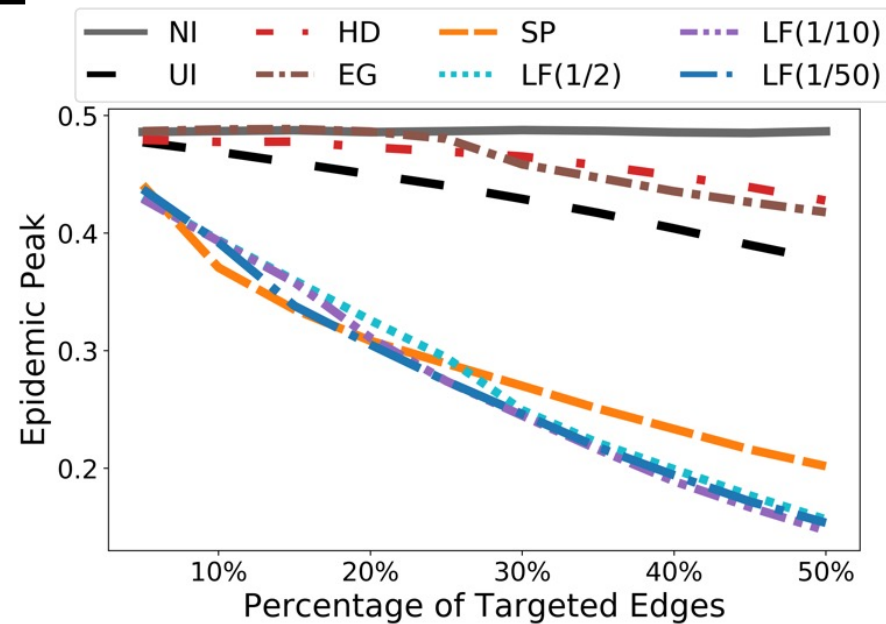**F**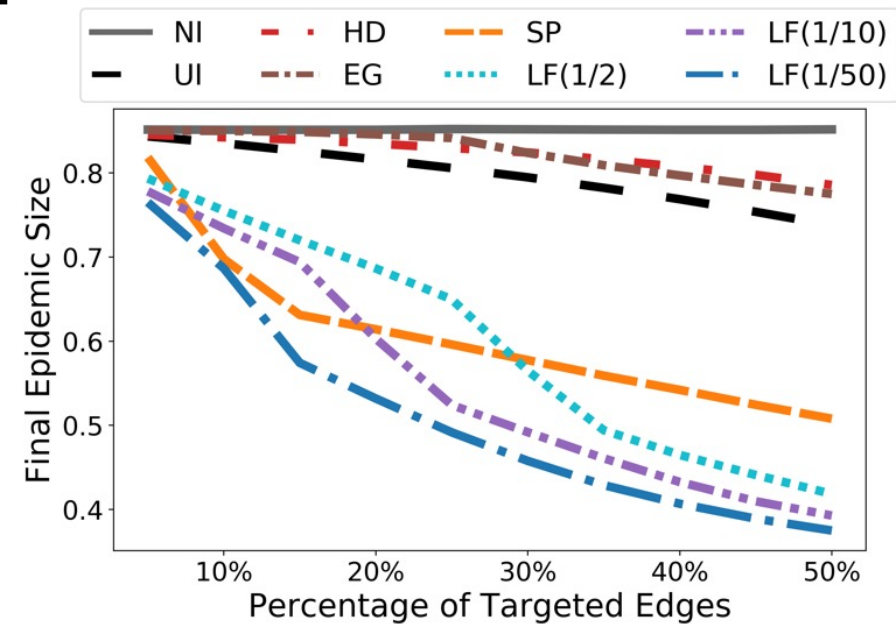

Supplement: S2 Fig — We illustrate predicted epidemic curves, epidemic peaks and epidemic sizes for two different initialization scenarios. We plot epidemic curves at 25% intervention coverage level. We average over 50 trials for random initialization. LF is shown to be the most effective at reducing both epidemic peaks and total outbreak sizes. (A)-(C) The epidemic starts from a well-connected cluster of 101 infected persons. (D)-(F) The epidemic starts from a random selection of 0.1% of all population as initially infectious. (PDF) [file pcbi.1009351.s004.pdf]

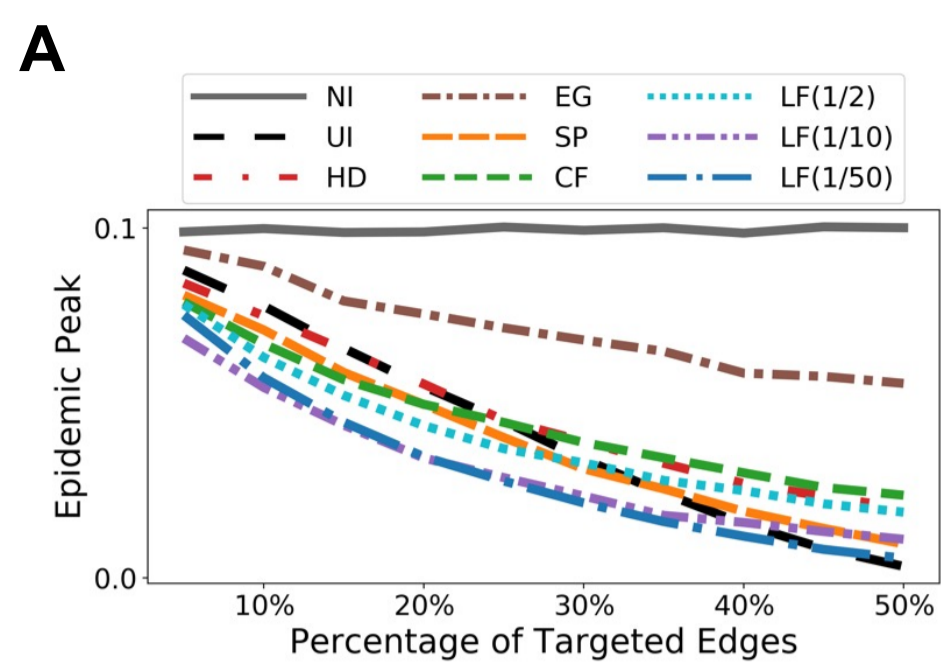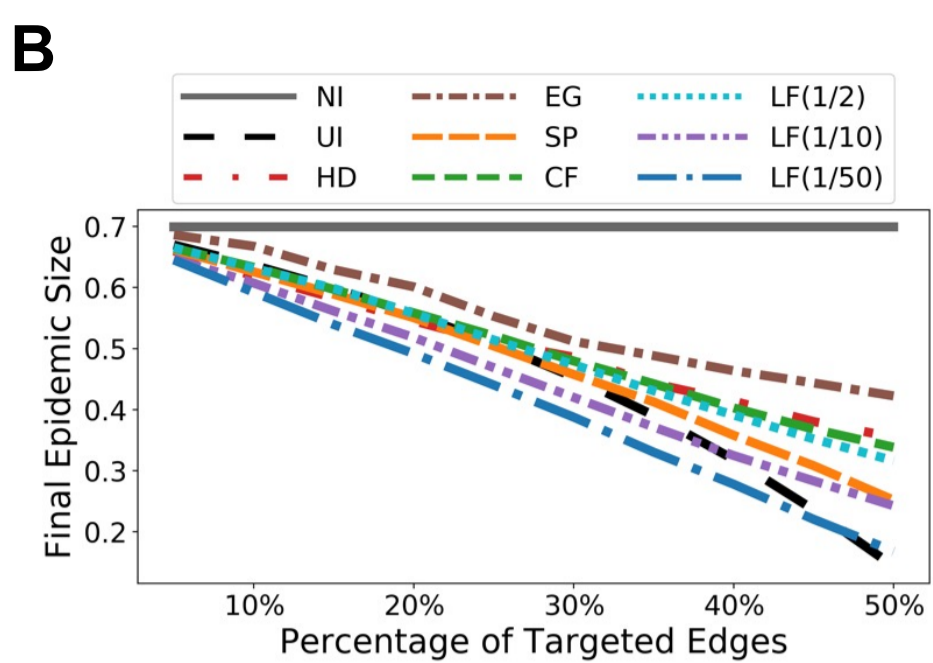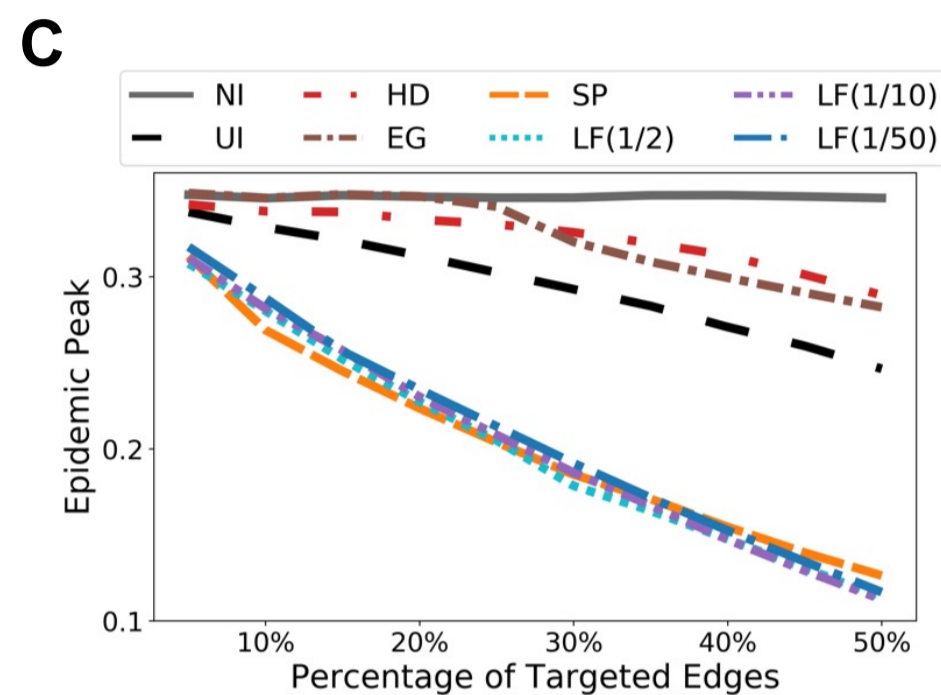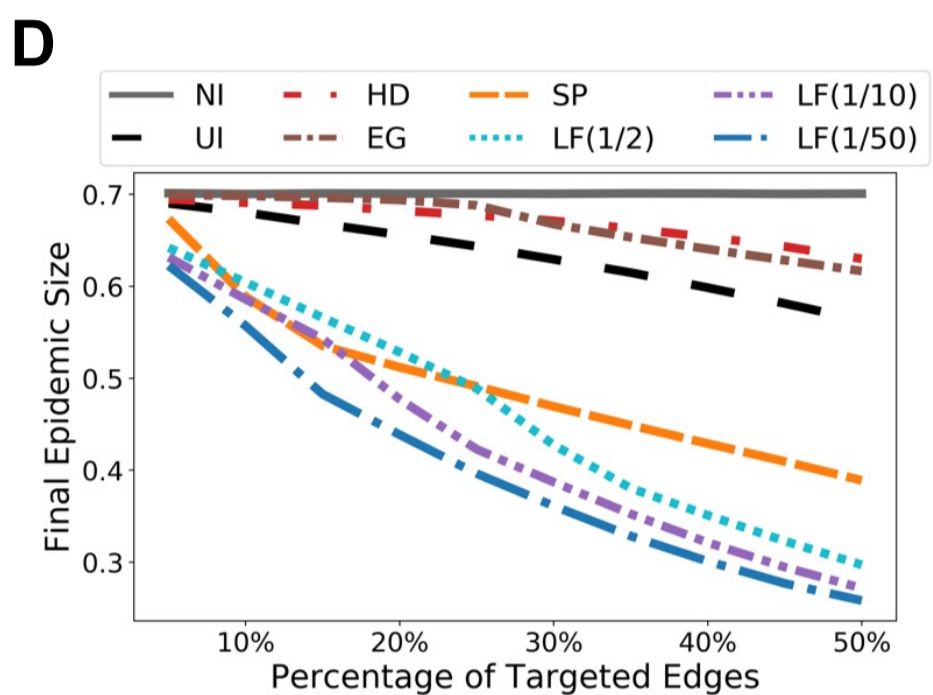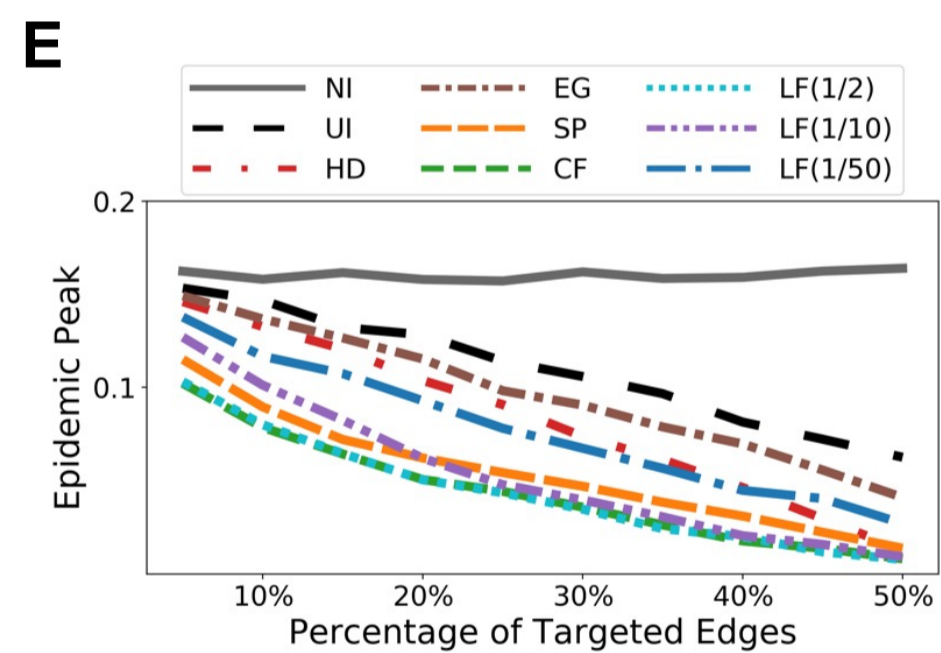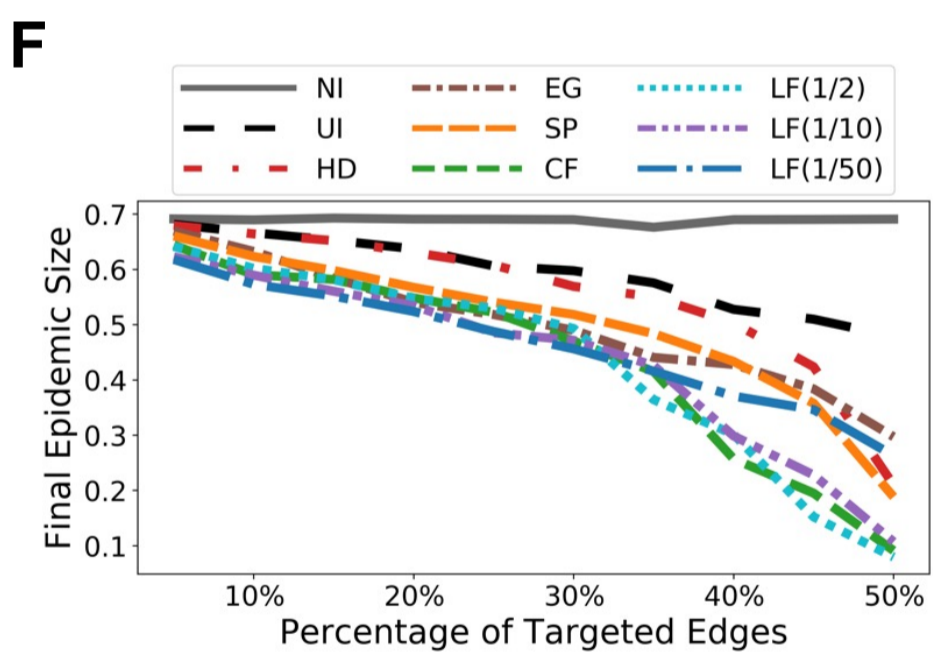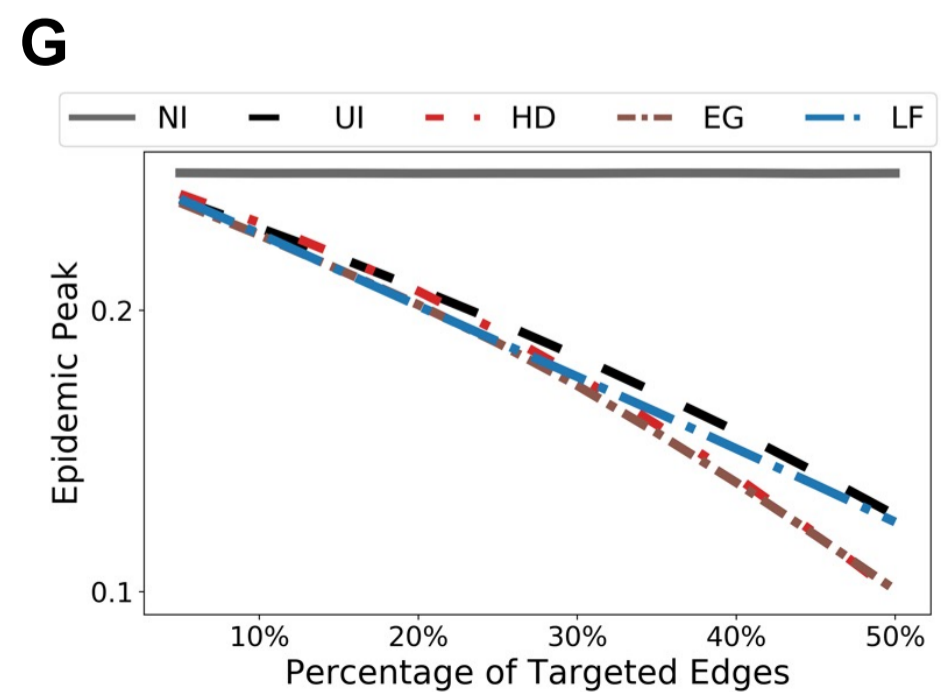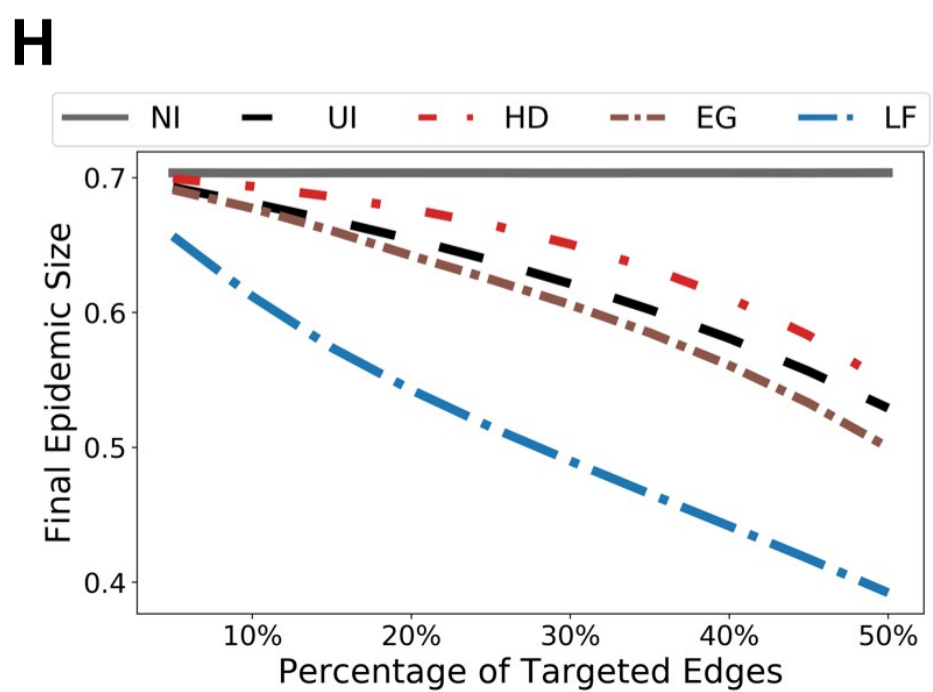

Supplement: S4 Fig — All other model parameters and intervention settings are the same. We show simulation results from random initialization of the SEIR models. Other model initializations lead to the same conclusion. LF intervention still delivers the best overall performance. (A)-(B) Results for Facebook County. (C)-(D) Results for Wi-Fi Montreal. (E)-(F) Results for sub-sampled Portland network. (G)-(H) Results for full Portland network. (PDF) [file pcbi.1009351.s006.pdf]

**A**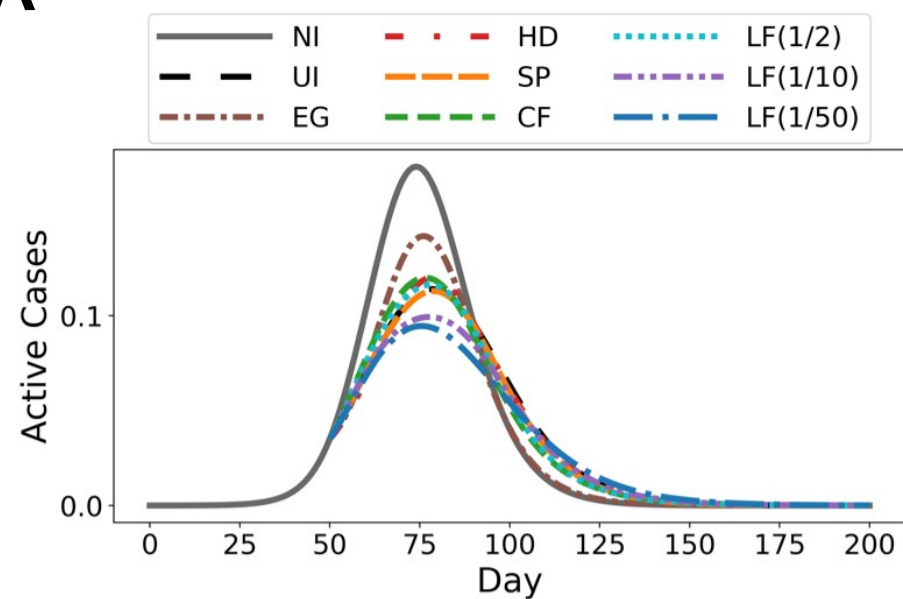**B**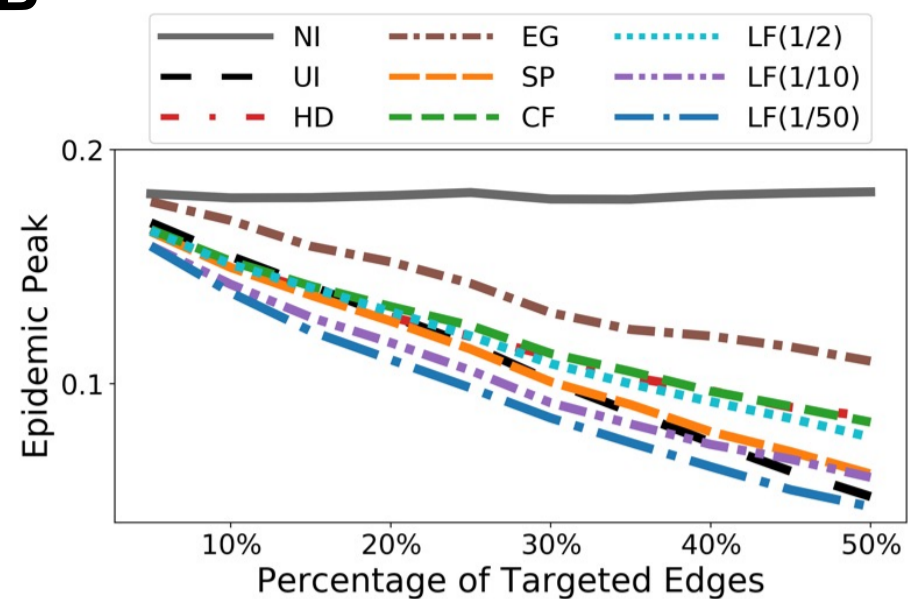**C**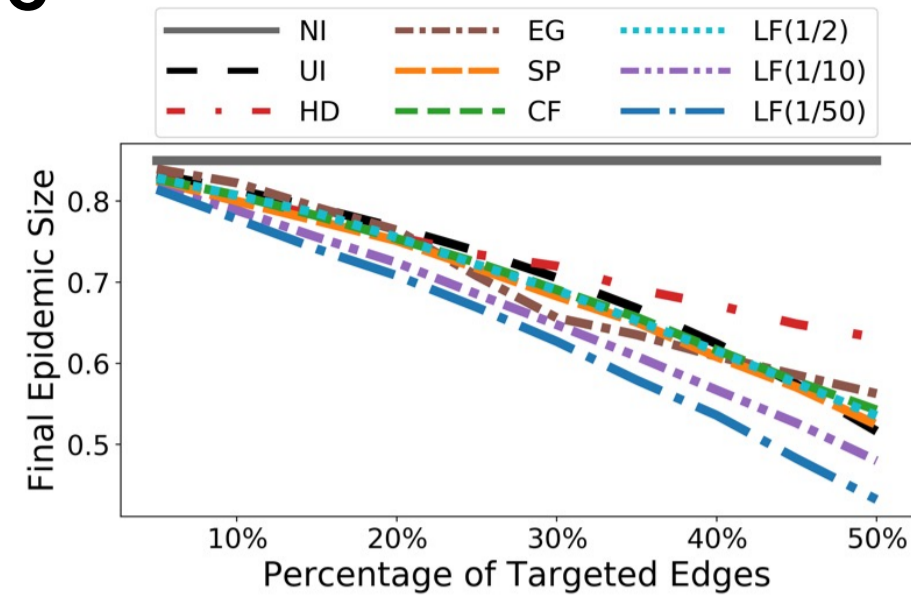**D**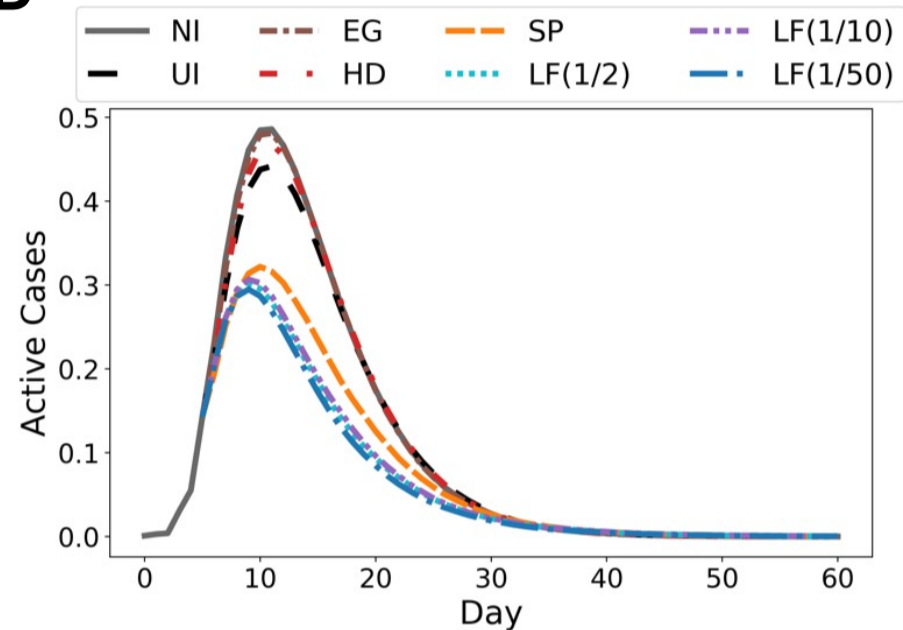**E**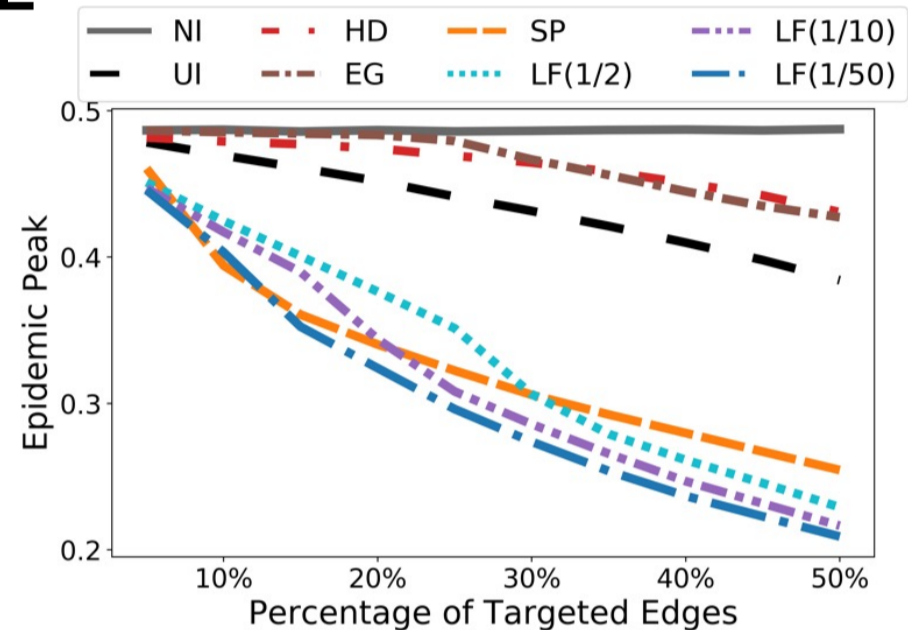**F**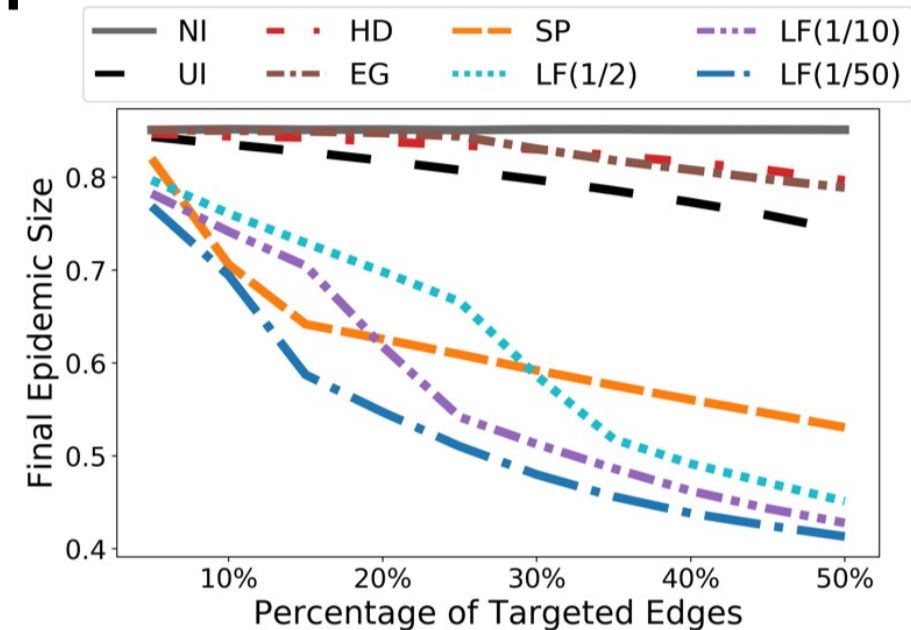**G**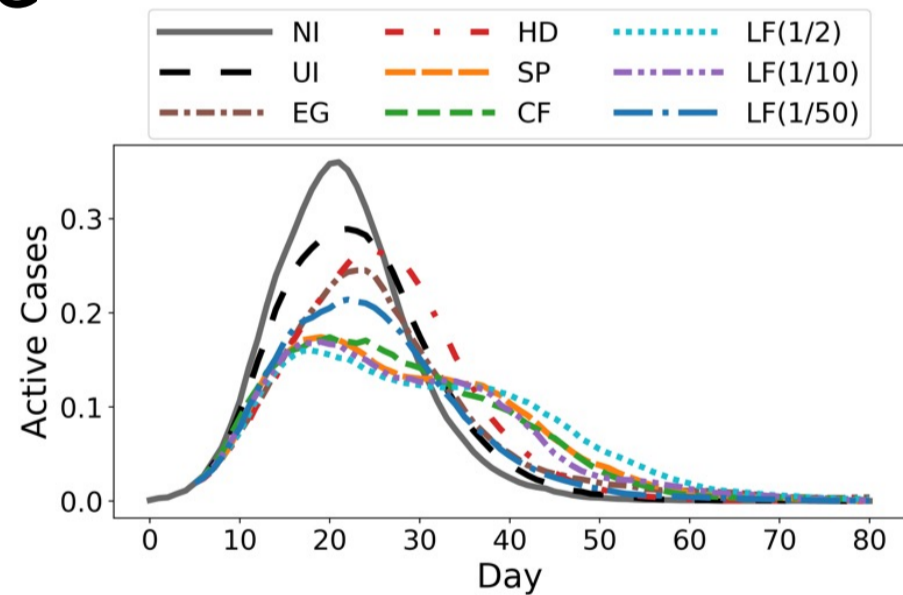**H**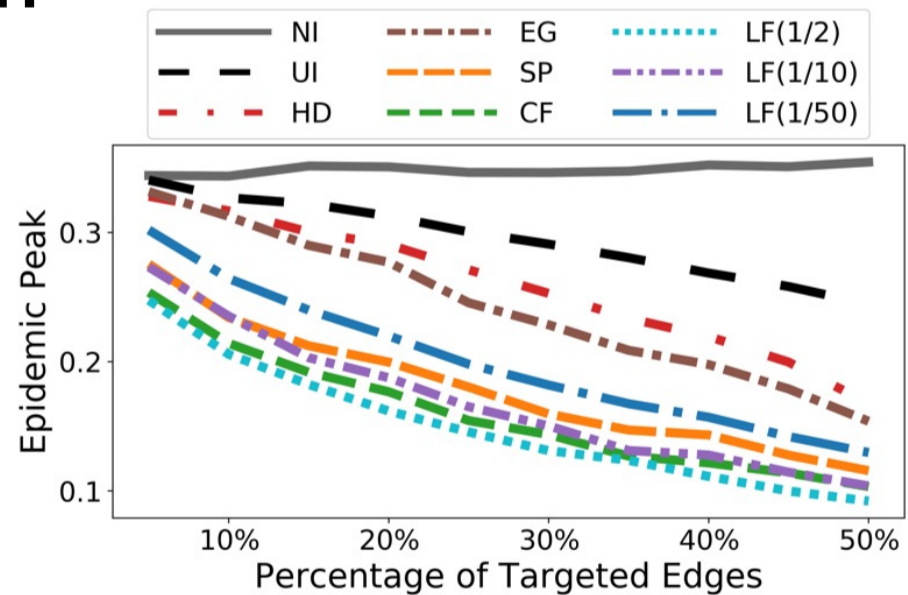**I**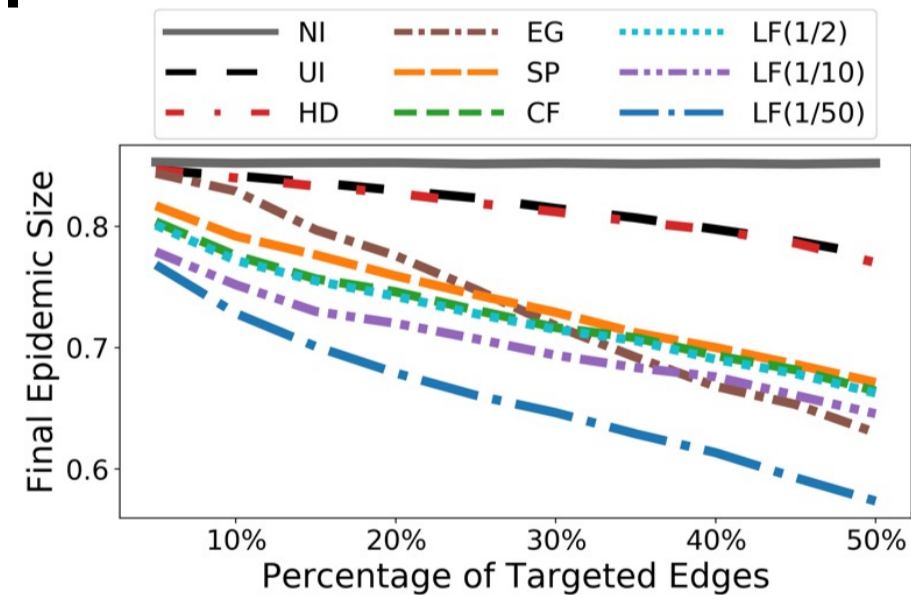**J**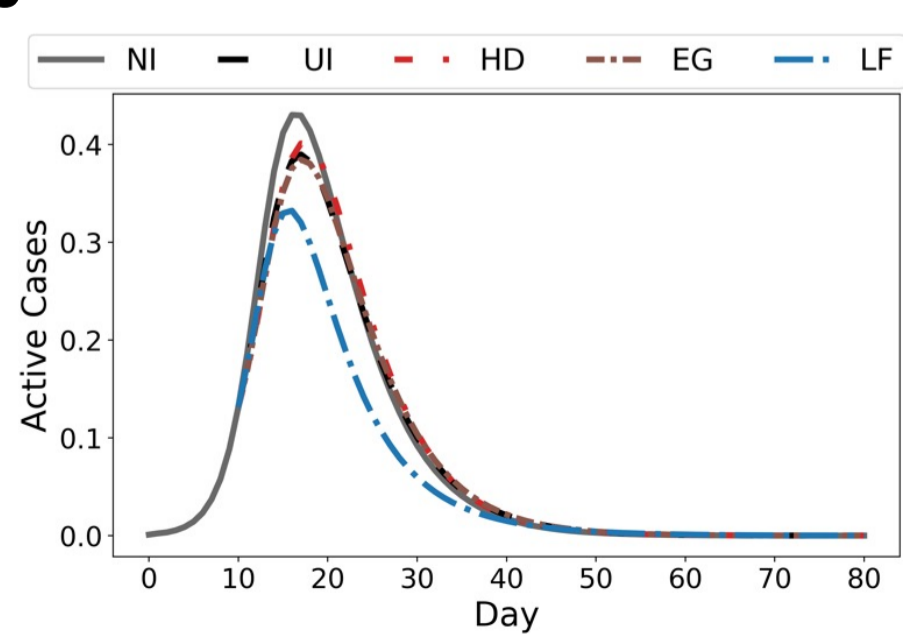**K**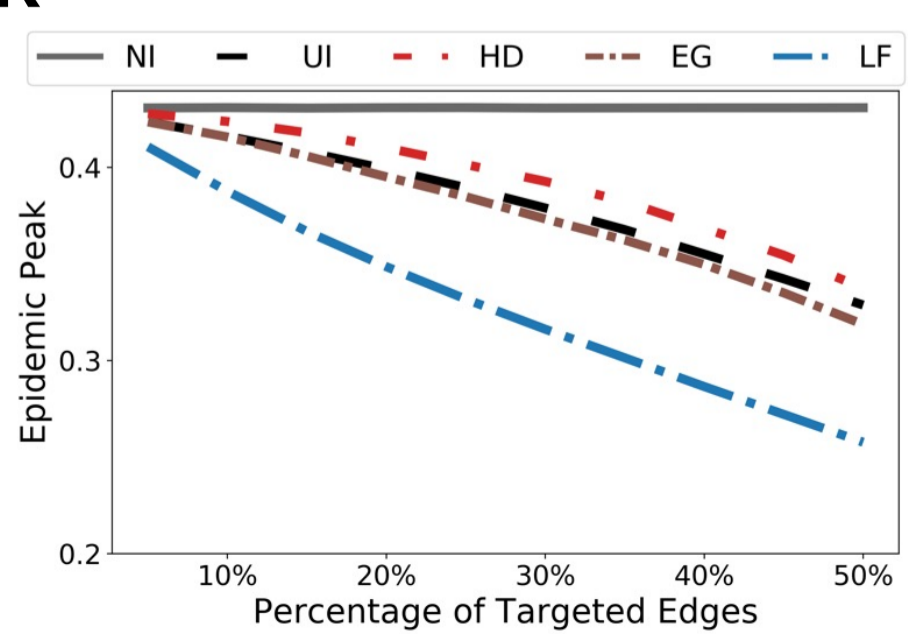**L**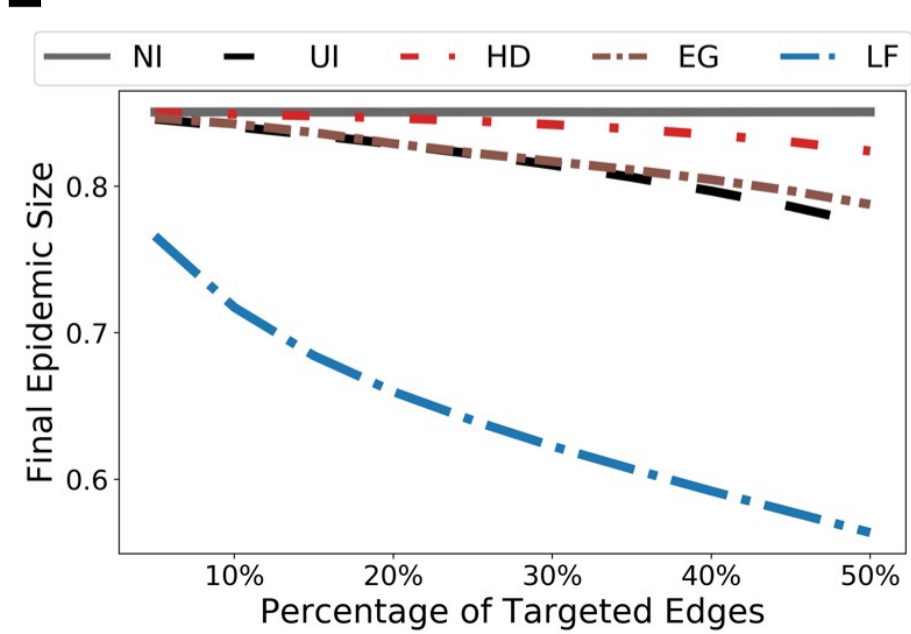

Supplement: S5 Fig — All other model parameters and intervention settings are the same. We plot epidemic curves at 25% intervention coverage level to illustrate intervention effects that start in the middle. We show simulation results from random initialization of the SEIR models. Other model initializations lead to the same conclusion. LF intervention still delivers the best overall performance. (A)-(C) Results for Facebook County. (D)-(F) Results for Wi-Fi Montreal. (G)-(I) Results for sub-sampled Portland network. (J)-(L) Results for full Portland network. (PDF) [file pcbi.1009351.s007.pdf]

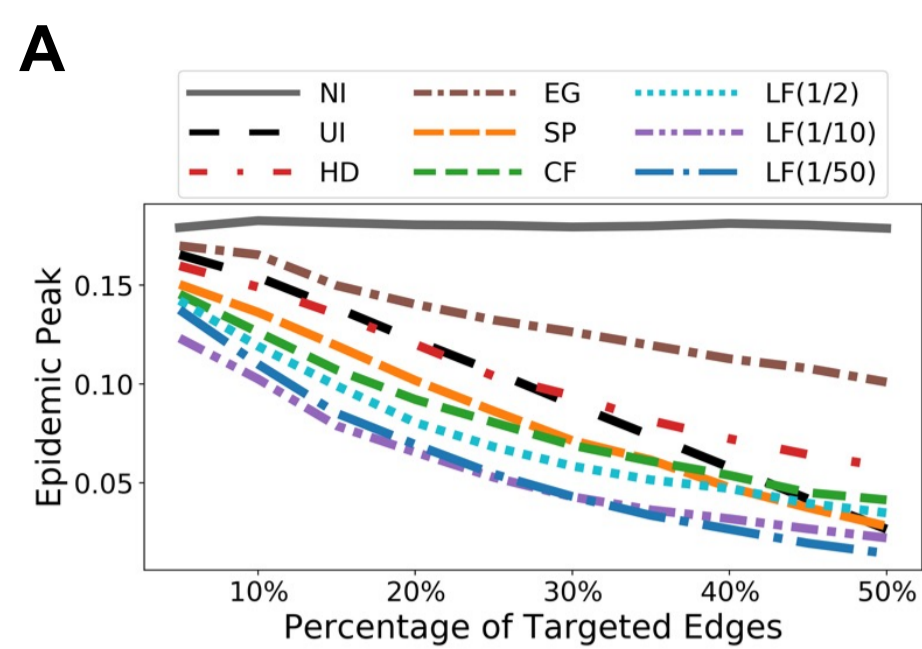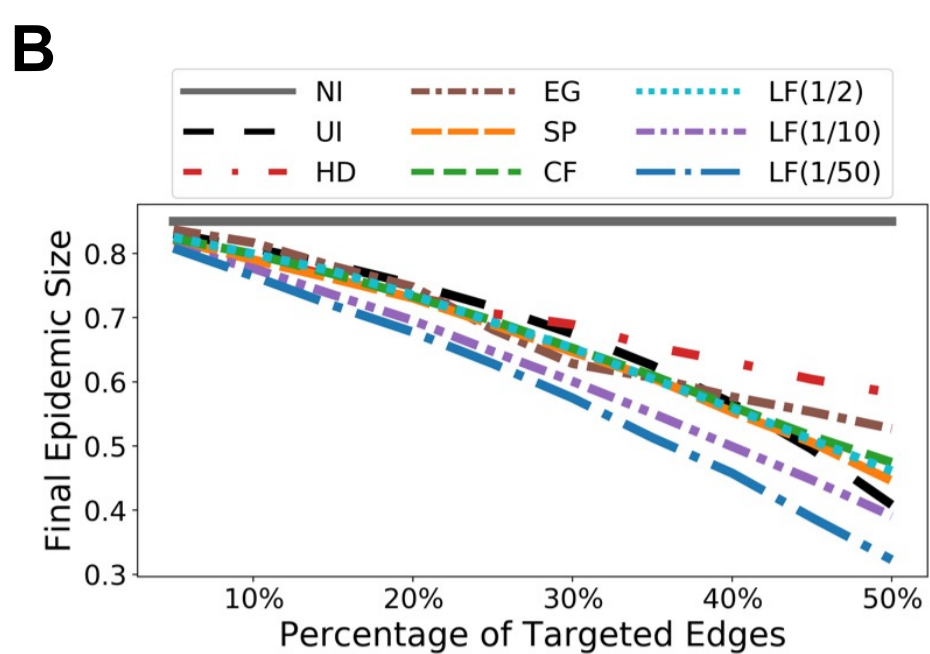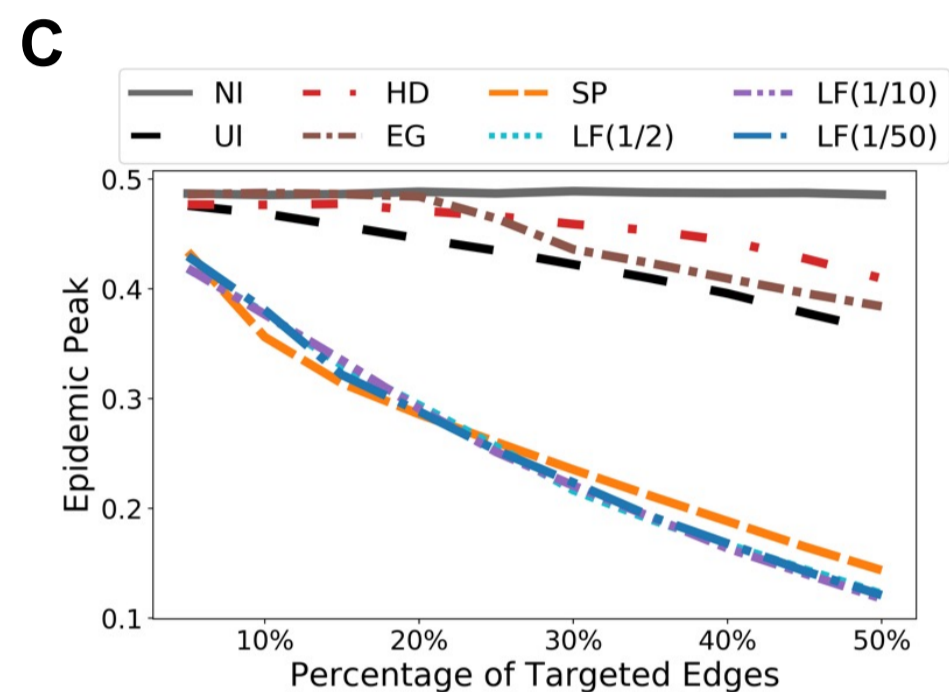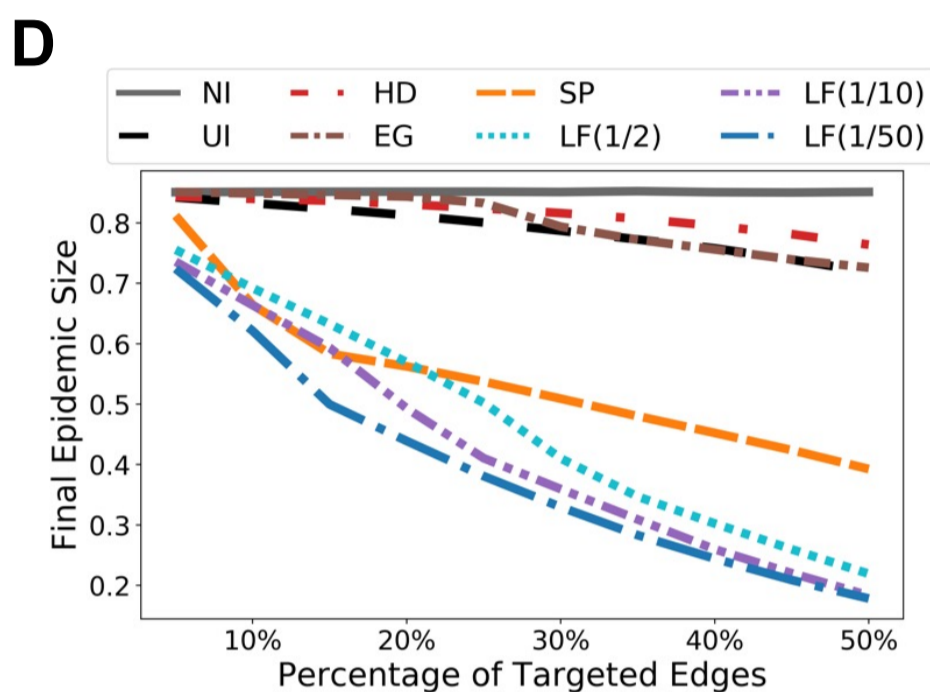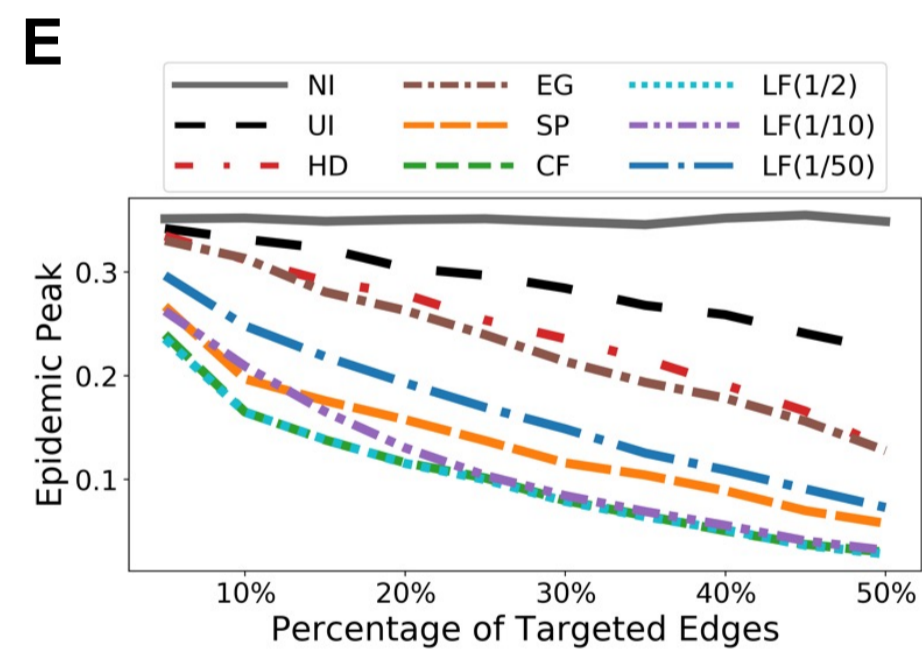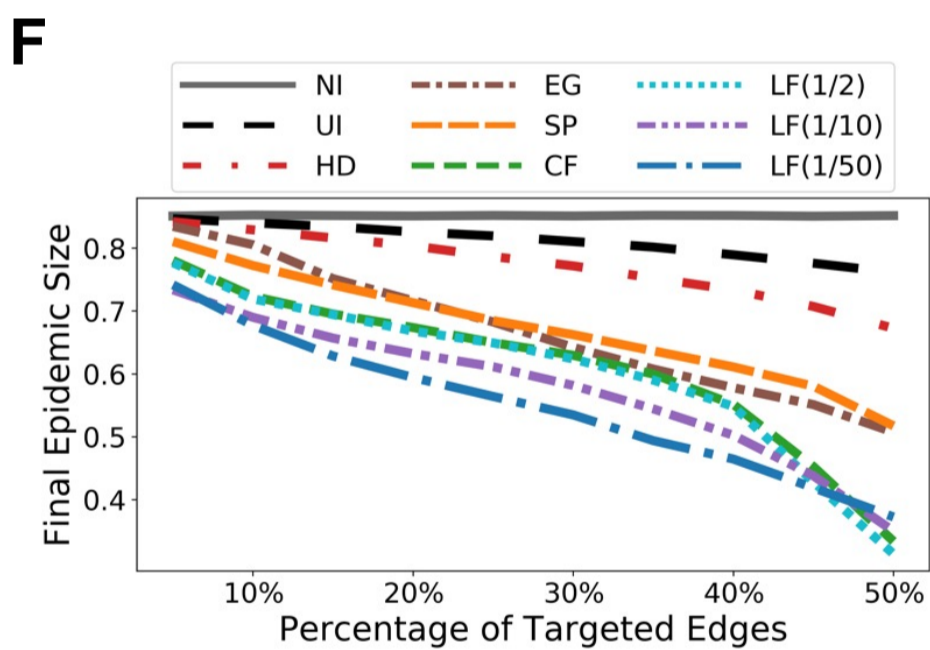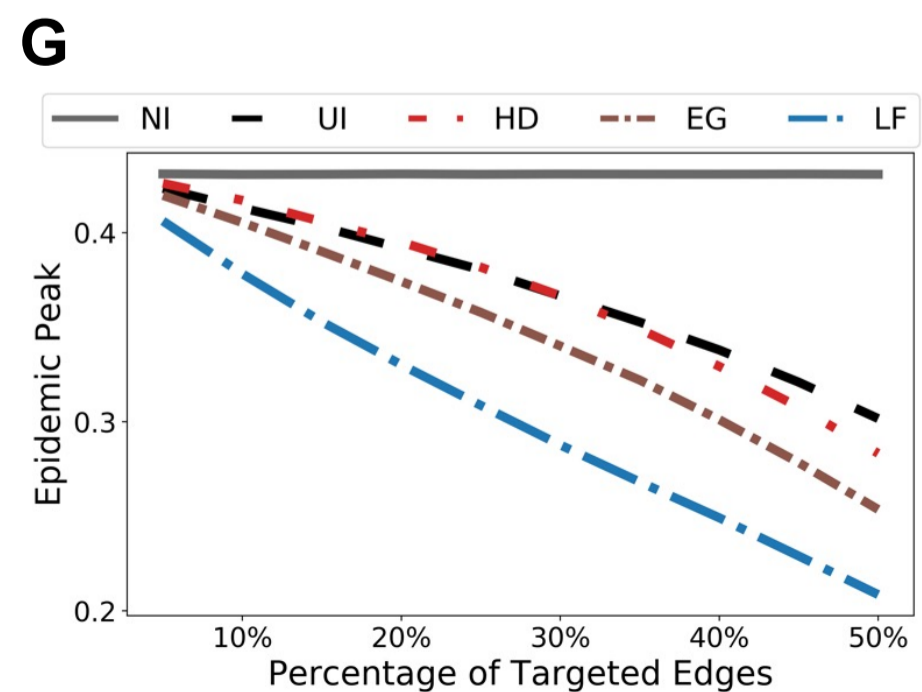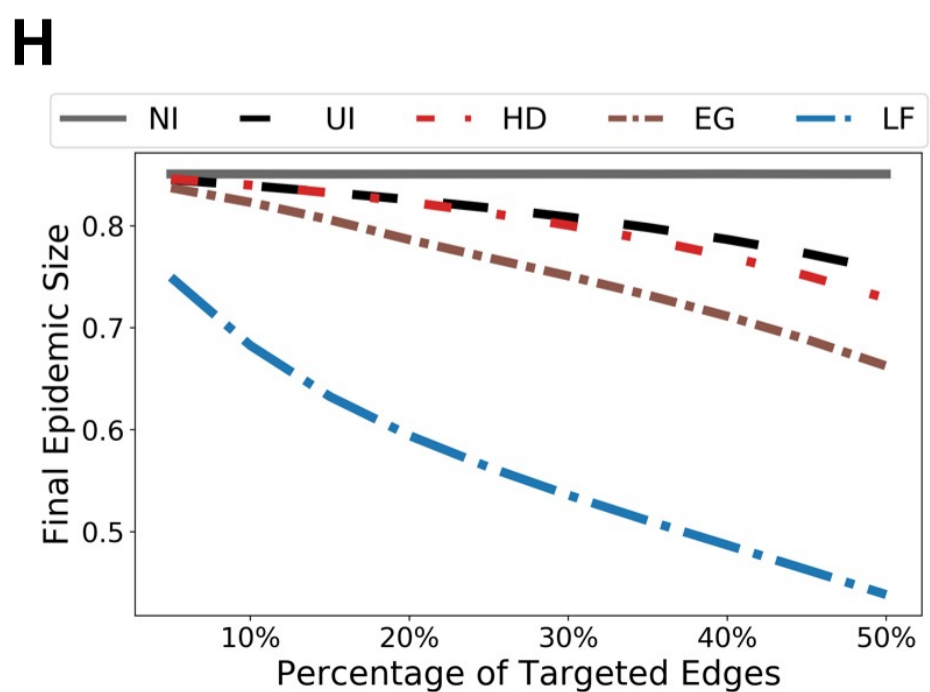

Supplement: S6 Fig — All other model parameters and intervention settings are the same. We show simulation results from random initialization of the SEIR models. Other model initializations lead to the same conclusion. LF intervention still delivers the best overall performance. (A)-(B) Results for Facebook County. (C)-(D) Results for Wi-Fi Montreal. (E)-(F) Results for sub-sampled Portland network. (G)-(H) Results for full Portland network. (PDF) [file pcbi.1009351.s008.pdf]

**A**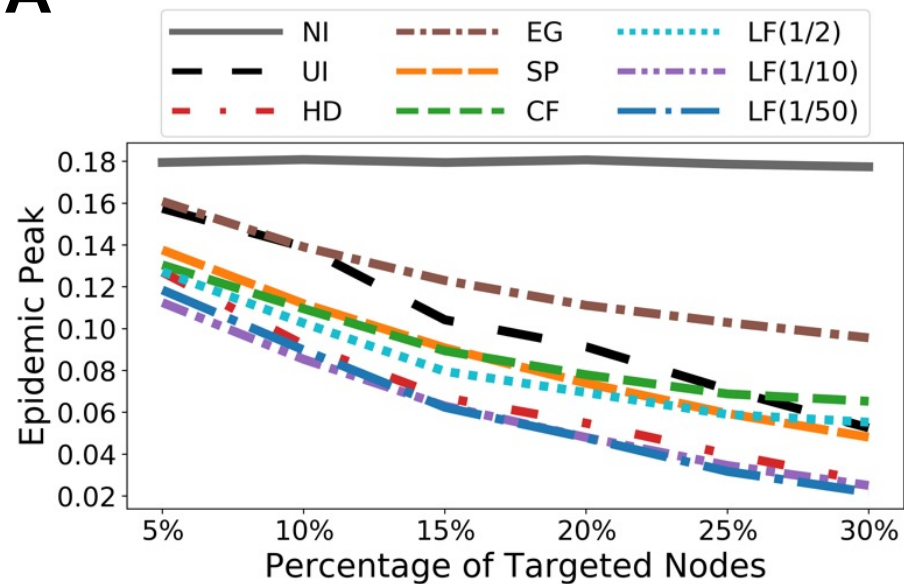**B**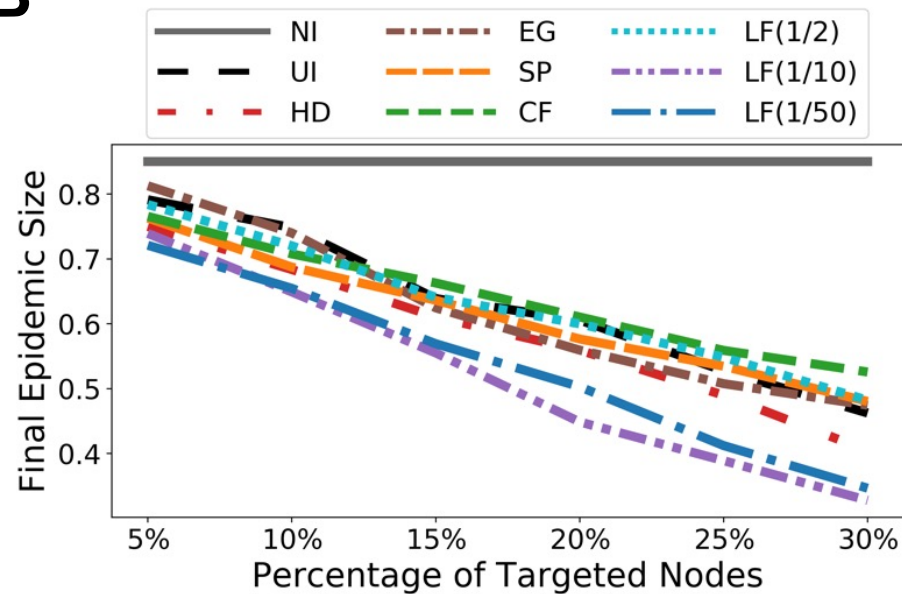**C**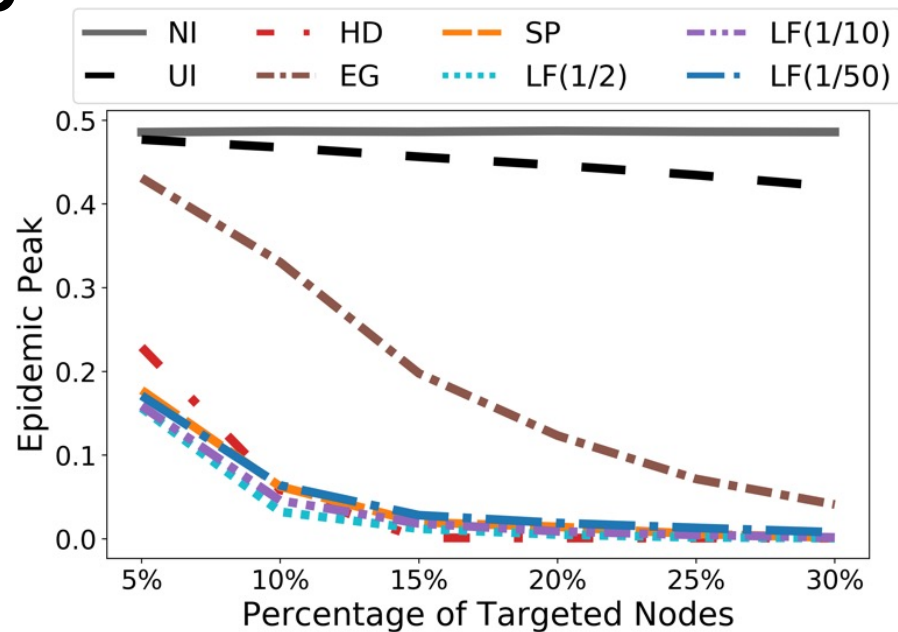**D**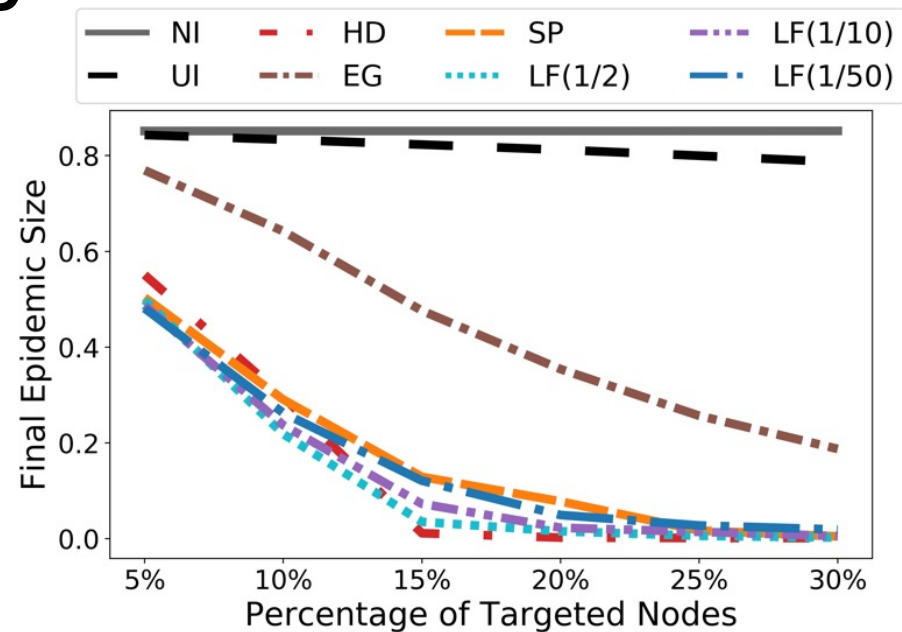

Supplement: S7 Fig — (A)-(B) Results for Facebook County. Node selection method based on LF betweenness gives the most reduction in both epidemic peaks and sizes, at all levels of node coverage. (C)-(D) Results for Wi-Fi Montreal. LF is the most effective when the node coverage is less than 10%. (PDF) [file pcbi.1009351.s009.pdf]

**A**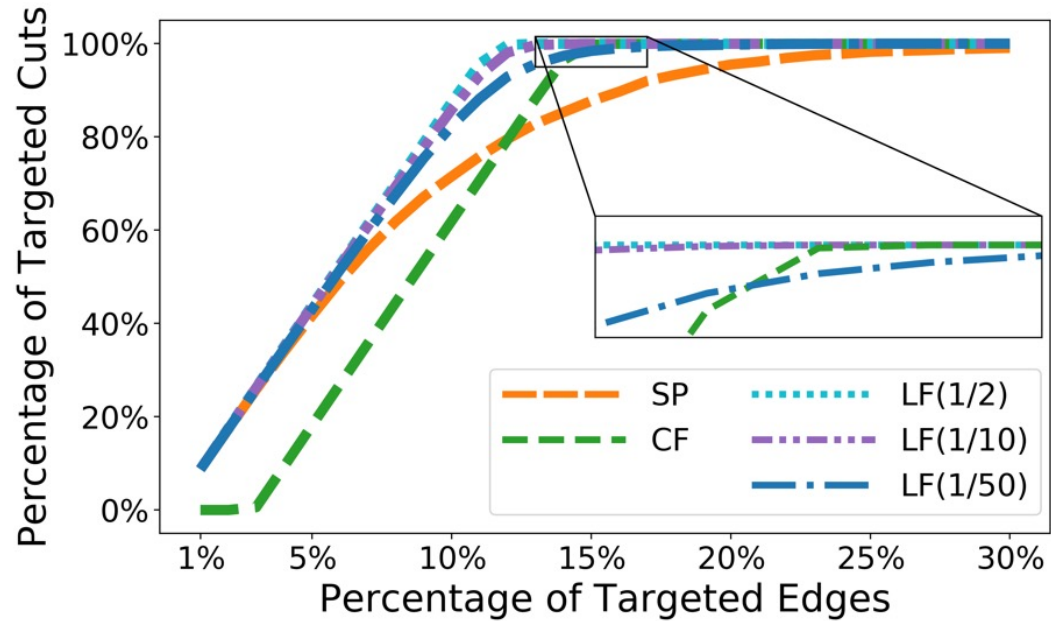**B**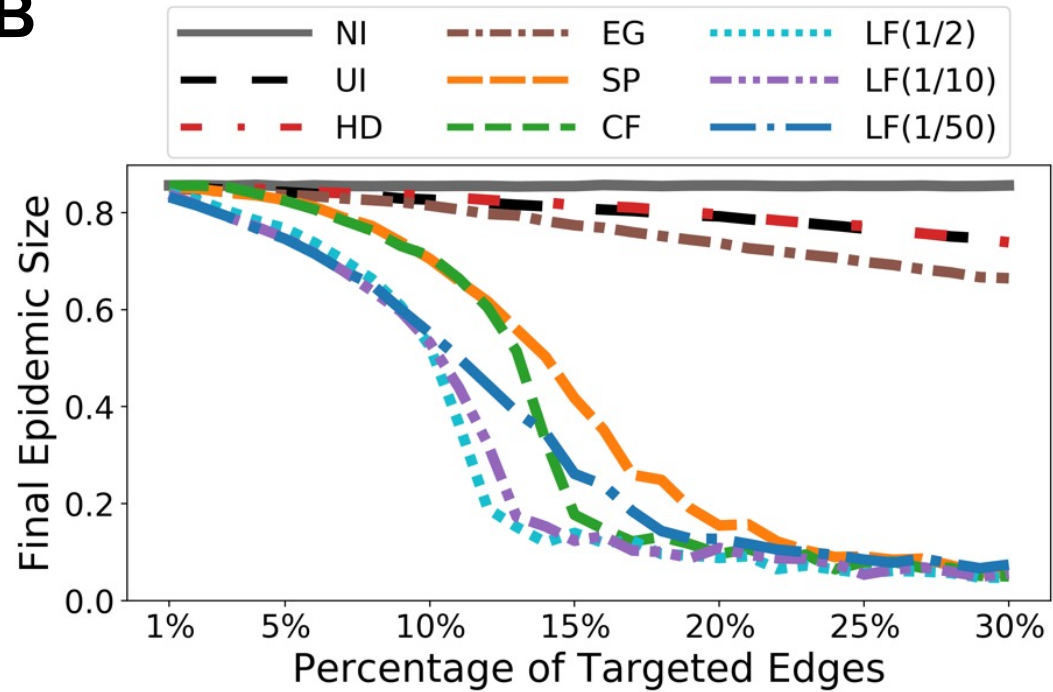

Supplement: S8 Fig — (A) Percentage of “cut” edges targeted by different betweenness measures with varying percentage of targeted edges. (B) Final epidemic sizes under different intervention strategies. We calibrated β so that 85% of the population would be affected without any intervention. All other model parameters and intervention settings are the same. We used random initialization of the agent-based SEIR model and we averaged over 50 trials to obtain the results in (B). Observe the close relationship between the number of targeted “cut” edges and the final epidemic sizes: The more “cut” edges targeted, the more effective the intervention strategy is. Overall, LF is the most effective at identifying the “cut” edges, which in turn helps produce the most effective interventions. (PDF) [file pcbi.1009351.s010.pdf]

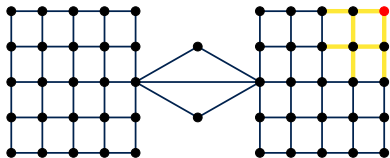

$\lambda = 0.1$

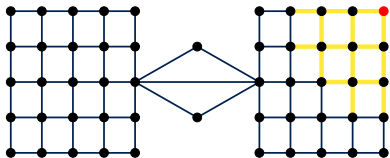

$\lambda = 0.2$

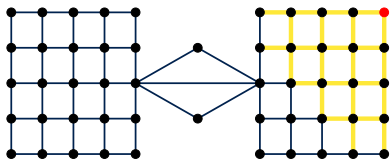

$\lambda = 0.3$

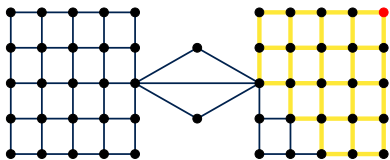

$\lambda = 0.4$

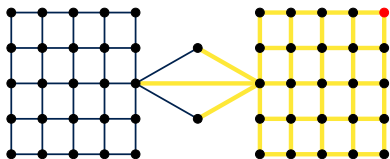

$\lambda = 0.5$

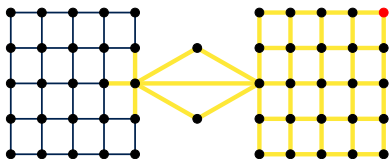

$\lambda = 0.6$

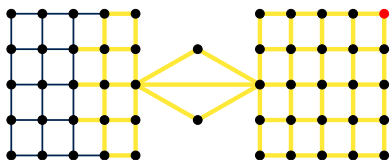

$\lambda = 0.7$

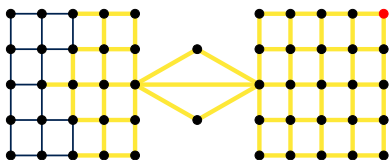

$\lambda = 0.8$

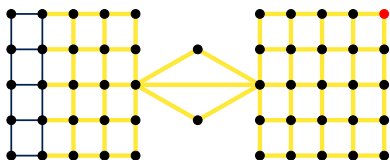

$\lambda = 0.9$

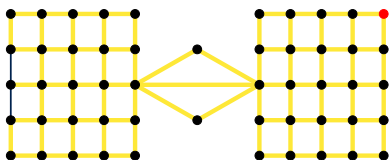

$\lambda = 1.0$

Supplement: S9 Fig — In each plot, the diffusion starts from the single source node coloured in red. Edges that have a nonzero flow crossing them are coloured in yellow. The plots show that as λ increases, the initial mass spread further away from the source node. When λ = 1, the initial mass are diffused to every node in the graph. (PDF) [file pcbi.1009351.s011.pdf]
