# Supplementary material for: Targeted pandemic containment through identifying local contact network bottlenecks
Source: PLoS Comput Biol. 2021 Aug 30;17(8):e1009351. doi: 10.1371/journal.pcbi.1009351 (PMC8432902; doi:10.1371/journal.pcbi.1009351)
Supplement: S1 Fig — We illustrate predicted epidemic curves, epidemic peaks and epidemic sizes for four different initialization scenarios. We plot all epidemic curves at 25% intervention coverage level. We average over 50 trials for random initialization. Observe that targeting edges according to LF betweenness leads to the most reduction in epidemic sizes for all initialization scenarios, and it consistently lowers the epidemic peaks by a large amount. Furthermore, even though EG has a good performance in terms of total epidemic sizes, it almost has no effect in reducing the epidemic peaks, making EG an undesirable method. (A)-(C) The epidemic starts from New York, New York. (D)-(F) The epidemic starts from Los Angeles, California. (G)-(I) The epidemic starts from a well-connected cluster of 67 counties. (J)-(L) The epidemic starts from a random selection of 1% of all counties across the country. (PDF) [file pcbi.1009351.s003.pdf]

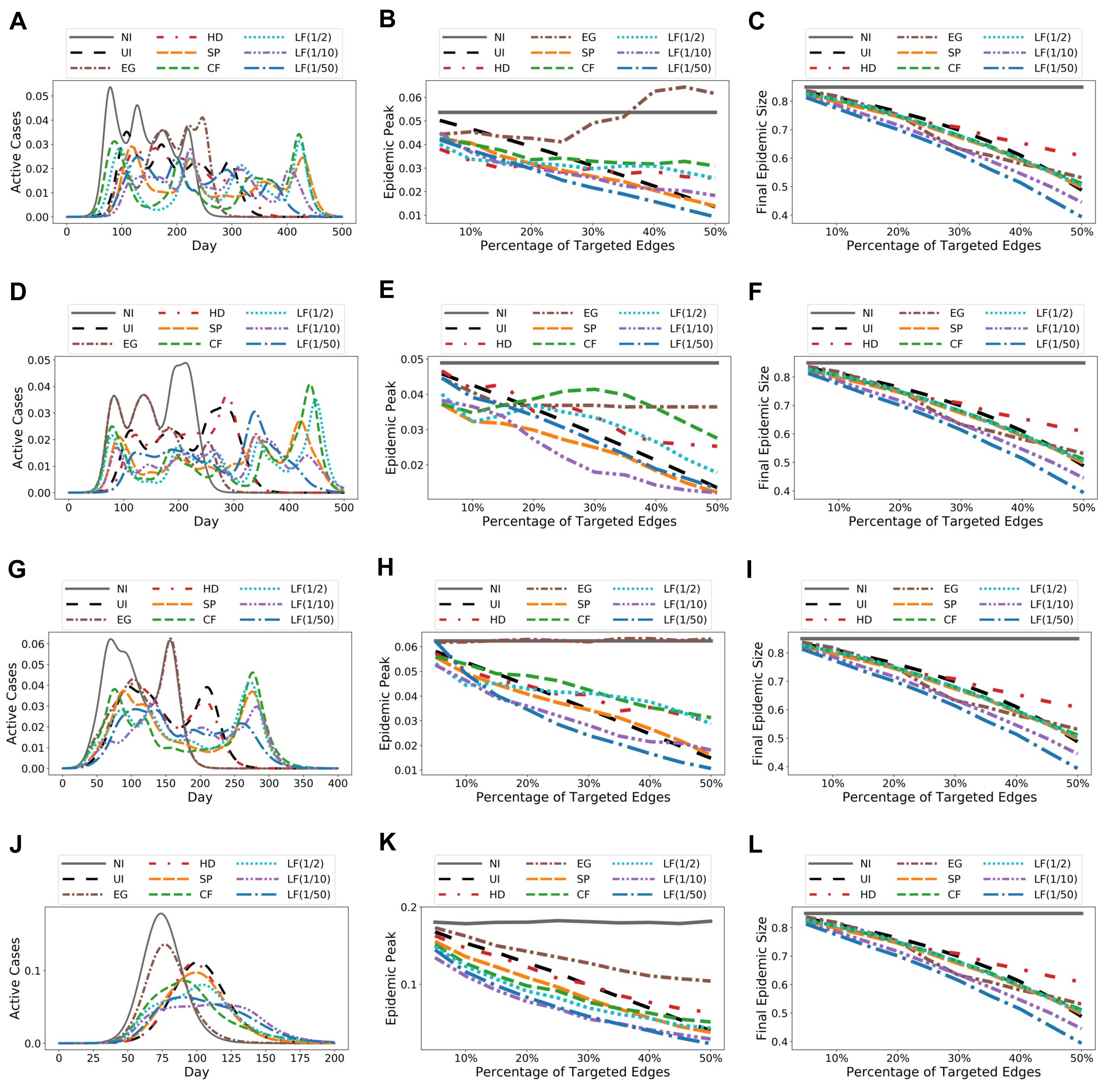
A
B
C
D
E
F
G
H
I
J
K
L
NI
UI
EG
HD
SP
CF
LF(1/2)
LF(1/10)
LF(1/50)
Active Cases
Day
Epidemic Peak
Final Epidemic Size
Percentage of Targeted Edges
