# Supplementary material for: Targeted pandemic containment through identifying local contact network bottlenecks
Source: PLoS Comput Biol. 2021 Aug 30;17(8):e1009351. doi: 10.1371/journal.pcbi.1009351 (PMC8432902; doi:10.1371/journal.pcbi.1009351)
Supplement: S3 Fig — We illustrate predicted epidemic curves, epidemic peaks and epidemic sizes for two different initialization scenarios. We plot epidemic curves at 25% intervention coverage level. We average over 50 trials for random initialization. (A)-(C) The epidemic starts from a well-connected cluster of 80 infected persons in the sub-sampled Portland network. (D)-(F) The epidemic starts from a random selection of 0.1% of all population in the sub-sampled Portland network as initially infectious. (G)-(I) The epidemic starts from a well-connected cluster of 37 infected persons in the full Portland network. (J)-(L) The epidemic starts from a random selection of 0.1% of all population in the full Portland network as initially infectious. (PDF) [file pcbi.1009351.s005.pdf]

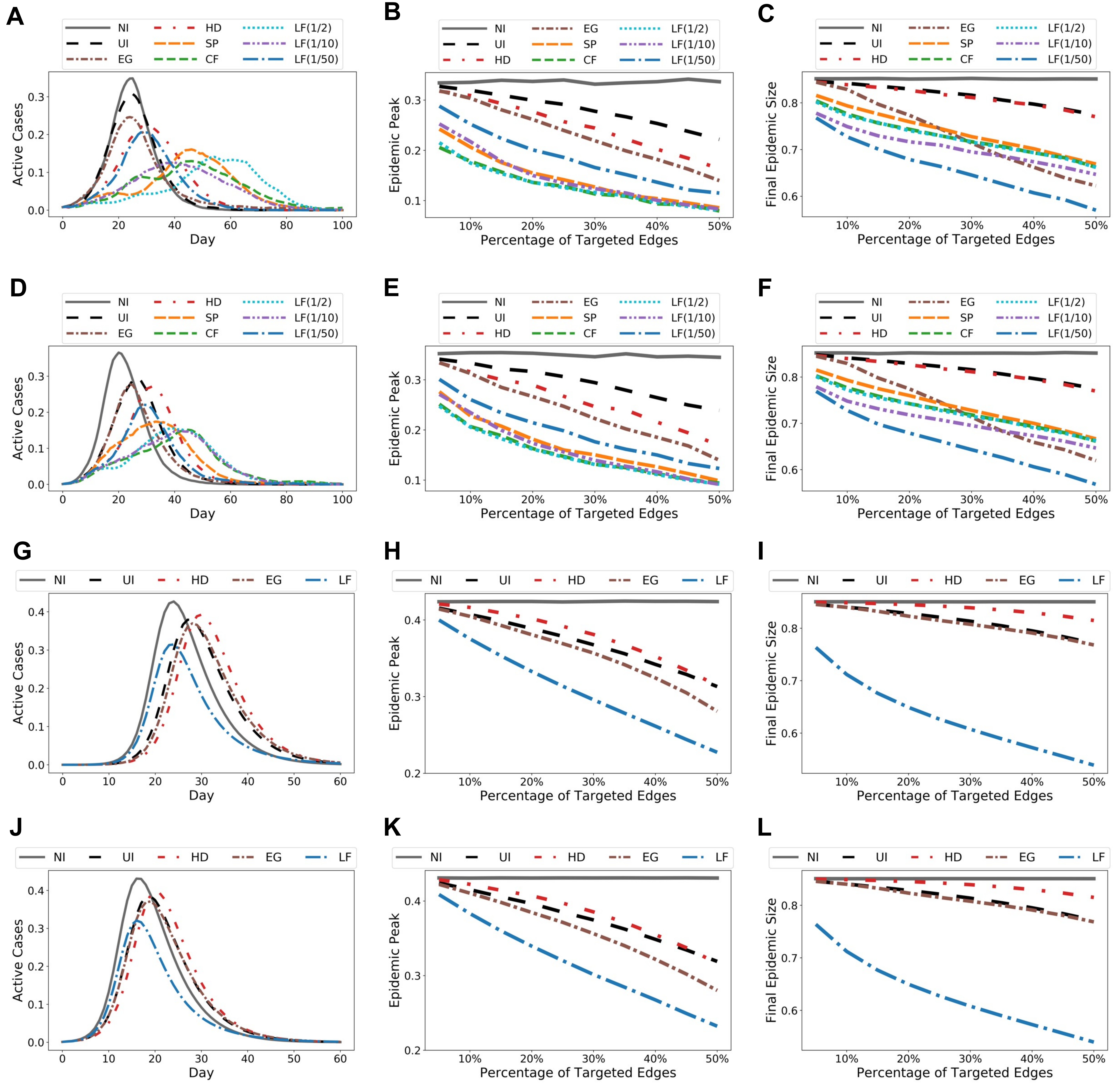

A
NI
UI
EG
HD
SP
CF
LF(1/2)
LF(1/10)
LF(1/50)
Active Cases
Day
B
Epidemic Peak
Percentage of Targeted Edges
C
Final Epidemic Size
Percentage of Targeted Edges
D
Active Cases
Day
E
Epidemic Peak
Percentage of Targeted Edges
F
Final Epidemic Size
Percentage of Targeted Edges
G
NI
UI
HD
EG
LF
Active Cases
Day
H
Epidemic Peak
Percentage of Targeted Edges
I
Final Epidemic Size
Percentage of Targeted Edges
J
Active Cases
Day
K
Epidemic Peak
Percentage of Targeted Edges
L
Final Epidemic Size
Percentage of Targeted Edges
