# Supplementary material for: Targeted pandemic containment through identifying local contact network bottlenecks
Source: PLoS Comput Biol. 2021 Aug 30;17(8):e1009351. doi: 10.1371/journal.pcbi.1009351 (PMC8432902; doi:10.1371/journal.pcbi.1009351)
Supplement: Attachment — Submitted filename: review_yang2021.pdf [file pcbi.1009351.s012.pdf]

# Info

Journal : PLOS Computational Biology

Title : Targeted Pandemic Containment Through Identifying Local Contact Network Bottlenecks

Authors : Shenghao Yang, Priyabrata Senapati, Di Wang, Chris T. Bauch, Kimon Fountoulakis

# Summary

The authors propose a new method to identify important edges for the transmission of contagions on contact networks, based upon a new flow-based edge-betweenness centrality measure (LF). This measure was introduced by 3 of the authors in a recent publication. They apply this method to two different contagion scenarios: in the first, nodes of the network represent subpopulations (counties in the US), and edges indicate if there is sufficient social interactions between counties. In the second, nodes are individuals and edges are interactions between individuals.

The authors find that their method is able to provide a more efficient targetting of important egdes when compared with other standard centrality measures (degree, eigenvector, betweenness, current-flow) in the different settings, as measured by 'maximal prevalence peak' and the total number of cases. They explain this result by the 'locality' and 'clustering' biases of the LF measure.

# General Comments

In general, the paper is interesting and carefully written. The authors have performed a broad numerical experiment and provide insights on how certain edges might be more important for transmission based on local features.

As far as I know, this study is original (being based upon a new measure), and could potentially inspire other works to improve measures for targeted interventions. However, the issues listed below must be addressed prior to publication.

# Major issues

- All experiments are parametrized such that without interventions, around 90% of the population would be affected (and even 95% in supplemental figure S5). Hence, none of their results illustrate the performance of their method in lower final size regimes. The way the authors arrive at such large total cases is also flawed: they want to have a basic reproduction number R0 of 2.5, but they use a formula for the final size Z = 1-e^(-ZR0) that is not appropriate for network contagion models in general (especially when the network is heterogeneous) [see Ref. 1,2]. In consequence, I believe the real R0 they use in their simulations is probably much higher than 2.5. They could parametrize their transmission probability beta using a more appropriate formula for R0, e.g., the one used in Ref. [3], but also I think it is important to validate their approach for various values of final size, not just 90%. In fact, it is possible that their measure might become less effective in these regimes: their method targets low degree nodes in the Montreal Wifi case study, but my intuition is that this strategy is only effective for a large final size.
- I am unsure if the focus on 'pandemic containment' is relevant and adequate. Even though their numerical experiment is calibrated using COVID19 parameters, I do not see a clear contribution for the ongoing (or future) pandemic response. Their method is not applied with the type of data (demographic, population movement, etc.) that they discuss in the introduction. I feel like it would be more appropriate to place the current work in a broader context, about 'epidemic' interventions.
- The authors present the 'betweenness methods' as state-of-the-art, but more sophisticated methods have been developed, building upon betweenness centrality measures for instance [4,5], or completely different approaches like this recent paper that uses the eigendrop of the largest eigenvalue of the non-backtracking matrix [6]. Overall, this paper must be better situated against the literature on targeted interventions. Since many approaches, builds upon betweenness centrality measures, the experiment and comparison done by the authors remain relevant, since their local-flow measure could be used to improve these more sophisticated approaches. But their contribution must be clarified.

# Minor Issues and Suggestions

- The concept of 'locality' at the beginning of the paper was vaguely expressed. It is not until further in the text that I finally grasped what was meant here. I suggest adding a sentence or two in the introduction to clarify the intended meaning.
- How is lambda chosen? Does the optimal lambda depends on the structure? As presented, it seems like lambda has been picked to provide the best results or chosen in an ad hoc way: a clear guideline for the choice of an appropriate lambda would be useful if someone wants to use the method.
- All experiments epidemic interventions were performed on some dataset, for which it is harder to disentangle the structural features. To illustrate the properties of the proposed method (clustering and locality biases), it would be clearer to add some tests with some standard random networks, e.g., Erdős–Rényi, Watts-Strogatz, etc.
- The captions are quite short, which makes it difficult to understand the figures. Please give a more in-depth explanation. Relevant parameters for the simulations should be found in the captions, or refer to the corresponding method section. In Figure 5, the first panel is completely omitted.
- For the two types of contagion model (population-based, individual-based) it should be stated what the interventions could represent, with how it could be implemented in practice.
- The method sections, especially those about the local-flow method, are quite difficult to follow without experience in local graph clustering. It uses a terminology which the readership of this journal might not be familiar with. It is quite difficult to infer why lambda controls locality and how the method induces local graph clustering bias from remarks 2 and 3.
- A brief mathematical definition and description of SP, CF and other methods should be provided in the method section. While reading, it was unclear to me how the ranking of edges with HD and EG was performed, since these are based on node properties: is an edge ranked according to the product of the centralities of the nodes at each endpoint, or the maximum, etc.?

# More specific comments

- Author summary: "localized network centrality measure" and "interventions that target globally" were not clear to me (prior to reading the paper).
- Line 16: "invasion threshold can change in networks", what is meant by this? That the structure affects the epidemic threshold?

- Line 43-45: the use of local and global is again not very clear in the context.
- Line 123: the correspondence of nodes/edges is missing for the Portland dataset.
- Line 132: "the lower the NCP is better", what is better? Please clarify.
- Line 173: "Both methods [...] dispersed or globally clustered". I am not sure I understand what this means when looking at figure 4.
- Caption Figure 3: lambda = a, I think this is a typo. Also "EG intervention does not reduce peak prevalence", do you have an explanation for that? This would improve the discussion.
- Line 238-240: Typo, it should be figure 9, not 8.
- Line 305: please describe one-hot vector.
- Line 329: I don't think the "dual problem" has been properly introduced.
- Line 330: is d with a bar the average degree? It has not been defined.
- In the algorithm 1, in the while loop, is the line r(u) <- 0 correct? Or should it be r(w) <- 0?
- Line 374-375: Aji is expressed as the "number of contacts", but for the Facebook county dataset, there is an edge if there is strong social interaction (from the dataset section). Please clarify.
- After line 449: for phi(S), I don't recall a definition for the conductance.
- Line 460: It should be stated here that Localgraphclustering API from the original paper has been used all along, instead of just in the caption of figure 2.

## References


[1] Meyers, L. A., Pourbohloul, B., Newman, M. E., Skowronski, D. M., & Brunham, R. C. (2005). Network theory and SARS: predicting outbreak diversity. Journal of theoretical biology, 232(1), 71-81.

[2] Hébert-Dufresne, L., Althouse, B. M., Scarpino, S. V., & Allard, A. (2020). Beyond R0: Heterogeneity in secondary infections and probabilistic epidemic forecasting. medRxiv.

[3] Herrera, J. L., Srinivasan, R., Brownstein, J. S., Galvani, A. P., & Meyers, L. A. (2016). Disease surveillance on complex social networks. PLoS computational biology, 12(7), e1004928.

[4] Holme, P., Kim, B. J., Yoon, C. N., & Han, S. K. (2002). Attack vulnerability of complex networks. Physical review E, 65(5), 056109.

[5] Schneider, C. M., Mihaljev, T., Havlin, S., & Herrmann, H. J. (2011). Suppressing epidemics with a limited amount of immunization units. Physical Review E, 84(6), 061911.

[6] Torres, L., Chan, K. S., Tong, H., & Eliassi-Rad, T. (2020). Node Immunization with Non-backtracking Eigenvalues. arXiv preprint arXiv:2002.12309.
