# Supplementary material for: Targeted pandemic containment through identifying local contact network bottlenecks
Source: PLoS Comput Biol. 2021 Aug 30;17(8):e1009351. doi: 10.1371/journal.pcbi.1009351 (PMC8432902; doi:10.1371/journal.pcbi.1009351)
Supplement: Attachment — Submitted filename: PLOSCB_response_letter.pdf [file pcbi.1009351.s013.pdf]

Response letter to referee comments

We would like to sincerely thank the referees for carefully reading our manuscript and providing us with insightful comments. Many of the comments are very constructive and this encouraged us to make serious improvements to the manuscript in both content and presentation. The main changes are that (i) we re-ran all simulations with the suggested (and additional) parameter settings that attest the robustness of our methods; (ii) we added a new sub-section in Results that discusses the robustness of our method under different parameter or model settings; (iii) we added a Discussion sub-section in Results and we revised Conclusion in order to properly position our methods in the broader literature of targeted interventions. We detail below our responses to individual comments.

1 Response to Referee #1's comments

We thank the referee for the comments that have helped us to an improved version of the manuscript. The list below contains the issues raised in the review (in italics), followed by our response.

Major Issues

- *All experiments are parametrized such that without interventions, around 90% of the population would be affected (and even 95% in supplemental figure S5). Hence, none of their results illustrate the performance of their method in lower final size regimes. The way the authors arrive at such large total cases is also flawed: they want to have a basic reproduction number R0 of 2.5, but they use a formula for the final size Z = 1-e^(-ZR0) that is not appropriate for network contagion models in general (especially when the network is heterogeneous) [see Ref. 1,2]. In consequence, I believe the real R0 they use in their simulations is probably much higher than 2.5. They could parametrize their transmission probability beta using a more appropriate formula for R0, e.g., the one used in Ref. [3], but also I think it is important to validate their approach for various values of final size, not just 90%. In fact, it is possible that their measure might become less effective in these regimes: their method targets low degree nodes in the Montreal Wifi case study, but my intuition is that this strategy is only effective for a large final size.*

  Response: We re-ran all simulations based on calibrated β so that the final epidemic sizes without interventions are 55%, 70%, 85%, respectively, for each dataset. The largest final size, 85%, is based on the final size result on the Portland contact network when β is parameterized according to Ref. [3], i.e., $R_0 = \beta(\langle k^2\rangle - \langle k\rangle)/\langle k\rangle$, where $R_0 = 2.5$. Our new simulation results in the revision indicate that LF-based interventions are robust and overall the most effective, however the best λ value could change with different final sizes. For details about the new empirical results, please see the highlighted updates in the revised Results section. For details about model parameter settings, please see the revised sub-section describing COVID-19 model parametrization in Methods.

- *I am unsure if the focus on 'pandemic containment' is relevant and adequate. Even though their numerical experiment is calibrated using COVID19 parameters, I do not see a clear contribution for the ongoing (or future) pandemic response. Their method is not applied with the type of data (demographic, population movement, etc.) that they discuss in the introduction. I feel like it would be more appropriate to place the current work in a broader context, about 'epidemic' interventions.*

  Response: Thank you for pointing out this ambiguity. We consider interventions that remove edges from the network, rather than nodes. In our view, the removal of edges corresponds most closely to non-pharmaceutical interventions (NPIs) that reduce contacts (edges) but do not change the nature of nodes, such as imposing travel restrictions between two cities, or a susceptible individual adopting contact

precautions to prevent being exposed to an infected individual. In contrast, pharmaceutical interventions (PIs) such as vaccines and antiviral drugs change the nature of the nodes. Both NPIs and PIs are often employed in epidemic outbreaks for endemic infectious diseases. But for pandemics caused by novel emerging pathogens, NPIs (i.e. edge removal, perhaps even including all edges emanating from a given node) are often the only means of combatting the pathogen while PIs are under development. Hence, we have retained the emphasis on 'pandemic containment' in our revision, but we explain our reasoning more carefully in the Introduction and we note that these measures could also be applied to epidemic containment. We also specify the kinds of pandemic interventions that link removal might apply to, at the end of the second paragraph of the revised Results section. In a similar vein, in the revised sub-section describing our datasets, we explain our reasoning for using the Montreal WiFi and Facebook networks as a proxy for physical contact networks that might be changed by non-pharmaceutical interventions, while also clarifying that they are not intended to be fully representative of physical contacts in those populations. (The Portland Oregon contact network does not suffer from the same issue since it was designed as a physical contact network.).

- *The authors present the 'betweenness methods' as state-of-the-art, but more sophisticated methods have been developed, building upon betweenness centrality measures for instance [4,5], or completely different approaches like this recent paper that uses the eigendrop of the largest eigenvalue of the non-backtracking matrix [6]. Overall, this paper must be better situated against the literature on targeted interventions. Since many approaches, builds upon betweenness centrality measures, the experiment and comparison done by the authors remain relevant, since their local-flow measure could be used to improve these more sophisticated approaches. But their contribution must be clarified.*

  Response: Thank you for pointing us to the papers and more sophisticated intervention strategies. We added relevant references and dedicated a paragraph in the revised Results section in order to better position the proposed LF betweenness in the literature on targeted interventions. We revised the wording in Conclusion accordingly. Please note that [6] is an iterative node immunization strategy based on eigenvectors of the non-backtracking matrix, and thus it is not directly related to edge-level interventions that we are concerned about in this work: As our experiments show that eigenvector-based methods are not suitable for edge-level interventions.

Minor Issues and Suggestions

- *The concept of 'locality' at the beginning of the paper was vaguely expressed. It is not until further in the text that I finally grasped what was meant here. I suggest adding a sentence or two in the introduction to clarify the intended meaning.*

  Response: We added a few sentences with more intuitions and physical analogies to how 'far' information can spread in the network. Please see highlighted changes in the Introduction.

- *How is lambda chosen? Does the optimal lambda depends on the structure? As presented, it seems like lambda has been picked to provide the best results or chosen in an ad hoc way: a clear guideline for the choice of an appropriate lambda would be useful if someone wants to use the method.*

  Response: We added a paragraph in the revised Results section where we discussed it in more details. In general, the 'best' lambda can depend on the nature of networks (population-based or individual-based), specific datasets, and estimated model parameters. Therefore, for our method to deliver the best results in practice, we recommend picking a good lambda based on simulation results on the specific networks data at hand.

- *All experiments epidemic interventions were performed on some dataset, for which it is harder to disentangle the structural features. To illustrate the properties of the proposed method (clustering and locality biases), it would be clearer to add some tests with some standard random networks, e.g., Erdős– Rényi, Watts-Strogatz, etc.*

  Response: Thank you for suggesting additional tests on synthetic graphs. The most appropriate synthetic network for demonstrating locality and clustering bias is probably the LFR model, which is a standard

benchmark for testing community detection or local clustering algorithms. Using a LFR network generator, we produced a network with 10,000 nodes and 38,313 edges. Each node represents an individual and belongs to one of the 323 a priori known communities with varying sizes from 20 to 50. This is close to a real human contact network where small groups of individuals (families, friends, colleagues) constitute closely connected communities. Our results on the synthetic LFR network show that intervention effectiveness perfectly aligns with the number of inter-community edges that are targeted by different methods: LF identifies most inter-community edges and thus leads to the most effective intervention results. Please see S2 Text and S8 Fig for details.

- *The captions are quite short, which makes it difficult to understand the figures. Please give a more in-depth explanation. Relevant parameters for the simulations should be found in the captions, or refer to the corresponding method section. In Figure 5, the first panel is completely omitted.*

  Response: We added more explanations to the captions.

- *For the two types of contagion model (population-based, individual-based) it should be stated what the interventions could represent, with how it could be implemented in practice.*

  Response: We added the following sentence in Results. "For the population-based model, the interventions could represent selective road closure, travel screening, or quarantining towns and cities, as happened during the Wuhan COVID-19 outbreak for instance. For the individual-based model, the interventions could represent public health measures that advise or incentivize individuals who are connected to a bottleneck to practice physical distancing."

- *The method sections, especially those about the local-flow method, are quite difficult to follow without experience in local graph clustering. It uses a terminology which the readership of this journal might not be familiar with. It is quite difficult to infer why lambda controls locality and how the method induces local graph clustering bias from remarks 2 and 3.*

  Response: Thank you for the valuable feedback. We added two paragraphs in Methods where some explanations and intuitions about remarks 2 and 3 are provided. We added S9 Fig to demonstrate how lambda controls the locality of edge flows on a concrete toy example.

- *A brief mathematical definition and description of SP, CF and other methods should be provided in the method section. While reading, it was unclear to me how the ranking of edges with HD and EG was performed, since these are based on node properties: is an edge ranked according to the product of the centralities of the nodes at each endpoint, or the maximum, etc.?*

  Response: We added mathematical definition and description of SP, CF and other methods in the revised Methods section. For HD and EG, an edge is ranked according to the maximum of its incident node centralities. We made it clear by providing more details in Methods.

More specific comments

- *Author summary: "localized network centrality measure" and "interventions that target globally" were not clear to me (prior to reading the paper).*

  Response: We revised the author summary to make these terms appear more natural without knowing the details of the paper.

- *Line 16: "invasion threshold can change in networks", what is meant by this? That the structure affects the epidemic threshold?*
  Response: Correct. This has been clarified in the revision.

- *Line 43-45: the use of local and global is again not very clear in the context.*

  Response: We revised the sentence and provided concrete examples for what are considered as "local".

- *Line 123: the correspondence of nodes/edges is missing for the Portland dataset.*

Response: We added the following sentence based on the original reference. “Each individual person is a node and two persons are connected by an edge if they collocated at the same location during a short period of time.”

- *Line 132: "the lower the NCP is better", what is better? Please clarify.*

  Response: We rewrote the sentence as “lower NCP means more significant clustering patten in the network (see Methods for details)”.

- *Line 173: "Both methods [...] dispersed or globally clustered". I am not sure I understand what this means when looking at figure 4.*

  Response: We revised the sentence as “Therefore, both methods demonstrate a global pattern as the targets of SP are dispersed over the entire network and the targets of CF are clustered in the middle.” We added more descriptions in the caption of Figure 4.

- *Caption Figure 3: lambda = a, I think this is a typo. Also “EG intervention does not reduce peak prevalence”, do you have an explanation for that? This would improve the discussion.*

  Response: lambda = a was not a typo, however we revised the corresponding sentence in the caption to make it clearer. For the observation that EG does not reduce peak prevalence for Facebook County network, we added the following explanation in the caption: “This is likely due to the epidemic curve has two modes and EG mostly targets on edges connected to the counties where the first peak happens, e.g., Fig 3b shows that EG significantly reduces the first epidemic peak but has almost no effect on the second.”

- *Line 238-240: Typo, it should be figure 9, not 8.*

  Response: Fixed.

- *Line 305: please describe one-hot vector.*

  Response: We are sorry that we missed the definition. We renamed it as “indicator vector” which is more conventional in the relevant literature, and we provided a short mathematical description for it.

- *Line 329: I don’t think the "dual problem" has been properly introduced.*

  Response: We included a complete mathematical formulation for the dual problem in the revision.

- *Line 330: is d with a bar the average degree? It has not been defined.*

  Response: $\bar{d}$ is the maximum degree over a certain set of nodes (which is not necessarily the set of all nodes). We revised the wording so that the emphasize is that $\bar{d}$ is a small integer not larger than the maximum degree in the graph.

- *In the algorithm 1, in the while loop, is the line r(u) <- 0 correct? Or should it be r(w) <- 0?*

  Response: The line is correct. We can think of r(u) as the amount of residual mass on node u. In the while loop, for any node u that has positive residual mass, we proceed to remove the mass from node u by sending some mass to each neighboring node w. As a result, node u will be left with 0 residual mass, so r(u) <- 0.

- *Line 374-375: Aji is expressed as the “number of contacts”, but for the Facebook county dataset, there is an edge if there is strong social interaction (from the dataset section). Please clarify.*

  Response: We revised the description to make it clearer.

- *After line 449: for phi(S), I don’t recall a definition for the conductance.*

  Response: We added a pointer to Equation (3) where we defined the conductance.

- *Line 460: It should be stated here that Localgraphclustering API from the original paper has been used*

all along, instead of just in the caption of figure 2.

Response: We added this sentence at the end of the paragraph.

2 Response to Referee #2's comments

We would like to thank the referee for carefully reading our manuscript and for the positive feedback. The list below contains the issues raised in the review (in italics), followed by our response.

- *The authors mention computational considerations and the computational efficiency of the proposed LF over CF in several places in the manuscript (e.g., pg. 17). However, it would be beneficial to the reader if the authors included perhaps a table of CPU times to directly compare between the measures being tested in the simulation results.*

  Response: Figure 12 in the revision (or Figure 11 in the original manuscript) shows the computation time for SP, CF, LF on the three datasets. We measured wall clock time, however since we did not use multi-threading, it is roughly the same as CPU time. As shown in Figure 12, the computation time for LF scales linearly with λ for $\lambda \in [1/128, 1/2]$. Since the actual λ values we used for the experiments are included in this range, one can obtain accurate estimates on the computation time for all betweenness measures we used in the experiments. For example, for Port. Sub. and $\lambda = 1/50$, Figure 12 shows that computing LF is around 100 times faster than SP and around 1000 times faster than CF.

- *How is the optimal $f^*_{\Delta,T}$ (pg. 15) computed in practice? More detail is warranted.*

  Response: We added a more specific relation between primal optimal solution $f^*_{\Delta,T}$ and dual optimal solution $x^*_{\Delta,T}$ in the revised Methods section. Essentially, once we obtain a dual optimal solution $x^*_{\Delta,T}$ using the while loop in Algorithm 1, we can recover the primal optimal solution by $f^*_{\Delta,T} = -Bx^*_{\Delta,T}$.

- *The authors mention calibration of the transmission parameter β (pgs. 21-22) in order to meet the assumption of R0=2.5 – how exactly was the calibration performed? How sensitive are the model outputs (and the overall results) on the value of this parameter and the other model parameters (σ, γ)?*

  Response: This question is closely related to referee #1's question on the robustness of our methods. We have improved our simulation settings to meet referee #1's suggestions, where we now calibrate β to reach different levels of final epidemic size, and we show that our methods are robust to varying β values that lead to very different epidemic sizes without intervention. For details on model calibration please see the revised sub-section describing COVID-19 model parameterization in Methods.

- *Can the authors comment on how reasonable is it to assume constant parameter values for average transmission, duration, etc., at different locations throughout the network in the SEIR ODE model (pg. 20)? Were there additional simulations run in which these values were assumed to vary based on location?*

  Response: The average transmission rate is defined per edge. In a network, different locations have different local network structures (e.g., edge connectivity pattern), and as a result different locations will experience different epidemic dynamics even if the parameters are constant. In this sense, it is reasonable to assume constant parameter values at different locations, and let the contact networks structure determine how the spread of disease will vary at different locations.

- *Most of the figures are far too small to easily view / interpret without zooming in – consider making the plots larger in the final version of the manuscript for the ease of the reader.*

  Response: We are sorry for such an inconvenience. We will definitely make the figures larger when we submit the final figure files.
