# Supplementary material for: Targeted pandemic containment through identifying local contact network bottlenecks
Source: PLoS Comput Biol. 2021 Aug 30;17(8):e1009351. doi: 10.1371/journal.pcbi.1009351 (PMC8432902; doi:10.1371/journal.pcbi.1009351)
Supplement: Attachment — Submitted filename: second_review_yang2021.pdf [file pcbi.1009351.s014.pdf]

Info

Journal : PLOS Computational Biology

Title : Targeted Pandemic Containment Through Identifying Local Contact Network Bottlenecks

Authors : Shenghao Yang, Priyabrata Senapati, Di Wang, Chris T. Bauch, Kimon Fountoulakis

General Comments

The authors have addressed all my concerns. The additional comments in the introduction and result sections greatly improve and clarify the manuscript. The experiment with the LFR network is very interesting and showcases the properties of the new method they propose. I recommend this manuscript to be accepted.

A last remark: The clarifications in the method section helped, but it remains very technical. Even though it is not necessary to appreciate the results in the paper, I would suggest that the authors add further explanations or references to help the readers navigate this section. For instance, I appreciate the added definition of the dual problem, but it is not obvious to me how Eq.4 constitutes a dual problem to Eq.1, a reference would be needed.

Some remaining typos

line 45: typo, "a majority of [...]"

line 90: typo in figure 1 caption: "measure"

line 184: typo, "pattern"
