# Supplementary material for: Targeted pandemic containment through identifying local contact network bottlenecks
Source: PLoS Comput Biol. 2021 Aug 30;17(8):e1009351. doi: 10.1371/journal.pcbi.1009351 (PMC8432902; doi:10.1371/journal.pcbi.1009351)

Response letter to referee comments

We would like to sincerely thank the referees for reading our revised manuscript and providing us with more constructive comments. This encouraged us to make further improvements to the manuscript. The main changes are that (i) we improved the presentation of LF betweenness in Methods by adding further explanations/references and simplifying notations; (ii) we revised the Discussion sub-section in Results to accommodate Referee #2's comments. We detail below our responses to individual comments.

1 Response to Referee #1's comments

We thank the referee for the supportive and encouraging comments. Below we provide our response following each specific comment (in bold or italics).

General Comments

A last remark: The clarifications in the method section helped, but it remains very technical. Even though it is not necessary to appreciate the results in the paper, I would suggest that the authors add further explanations or references to help the readers navigate this section. For instance, I appreciate the added definition of the dual problem, but it is not obvious to me how Eq.4 constitutes a dual problem to Eq.1, a reference would be needed.

Response: Thank you for providing valuable feedback and assessment on our presentation of LF betweenness. We revised the method section with the following changes:

a) We removed unnecessary use of mathematical notations (e.g., signed incidence matrix B and ℓ_2-norm $\|f\|^2$) in the presentation and discussion of the optimization problem (1). The updated notations (with explanations) in the revised version are more intuitive.

b) We provided a more explicit formula for LF betweenness, which converts the expectation into an explicitly averaged sum. The final simplified formula for LF betweenness is now expressed in a very similar way to that of SP or CF betweenness.

c) We added more explanations about notations and provided further intuitions for both Problem (1) and the definition of LF betweenness.

d) We added a reference for the dual problem.

Some remaining typos [...]

Response: All fixed.

2 Response to Referee #2's comments

We thank the referee for the thoughtful follow-up questions and comments. The list below contains the questions raised in the review (in italics), followed by our response.

- *In a reply to Review #1 regarding the optimal way to choose lambda in practice on real data, the authors suggest to perform a grid search and then "[s]imply pick the λ value to gives the best simulated intervention performance" (pg. 17) - grid searches are generally computationally expensive, can the authors comment on this / any limitations of choosing lambda in this manner? Further, it seems that the results are sensitive to the choice lambda - can this be further discussed? Are the authors suggesting to*

run the grid search on the real data sets directly, or on simulated data first? This is not clear in the newly added paragraph on pg. 17. If the latter is meant, how does this connect to the real data?

Response: Yes, grid search can be computationally expensive, but that also depends on how coarse or fine the grid is. For most intervention purposes it would suffice to use a coarse grid with either linear or log scale. For example, in our experiments we mostly use $\lambda \in \{1/2, 1/10, 1/50\}$, and the simulation results reveal that $\lambda = 1/50$ is a good choice. In practice, the total computation time is also greatly affected by the size of the underlying contact network. For relative small networks, for example the population-based Facebook-County network which has 3100 nodes, computing LF betweenness takes only a few seconds to a few minutes, and thus it is practical to run the grid search using the original network. On the other hand, for much larger networks, for example the agent-based full Portland contact network which has more than 1.6 million nodes, it is more feasible to run the grid search using sub-sampled networks, with the expectation that if sub-sampling preserves important network structures of the full network, then a good λ value that works well on a sub-sampled network should also work well on the full network.
We can view the flexibility to pick λ for LF as an advantage, as there is no single measure that should work well for all settings. For example, if we compare SP and CF betweenness measures, sometimes SP is better than CF, sometimes CF is better than SP (e.g., see Fig. 10a and Fig. 10b), there is no consistent winner between the two. On the other hand, a nice property of LF is that we can control locality through λ, and our experiments show that for all settings (i.e., both population-based and individual-based models, different networks, different model parameters), there is a $\lambda \in \{1/2, 1/10, 1/50\}$ that outperforms all other methods. Indeed, intervention effectiveness can depend on the specific choice of λ. For epidemics that result in high final outbreak sizes without the presence of intervention, e.g., the COVID-19 pandemic, it is more important to isolate small-scale local communities, and thus a smaller $\lambda = 1/50$ is more effective. On the other hand, for epidemics that only affect a smaller fraction of the entire population without the presence of intervention, it may be more effective to focus on slightly more globally important bottleneck edges, and thus a larger $\lambda = 1/2$ may work better (e.g., see Fig. 10b). There are a number of complicating factors for epidemic dynamics over real networks, these include different network structures and different epidemic model parameters, so in practice we recommend picking a good λ based on simulation results using the specific setting of interest. Our goal in this work is demonstrating that localized measures are important for targeted pandemic containment, and we provide a flexible tool (i.e., LF betweenness) so that the users can both make decision about and have control over "how local" they should go based on their specific contexts.
We revised the Discussion sub-section in Results accordingly.

- *I am still not convinced why the authors assume the values for sigma_i and gamma_i are fixed across all locations, as done in this study (see pg. 25, line 624) - can the authors comment on this? Were additional simulations run in which these values varied by location?*

  Response: Thank you for following up on this. We are sorry that we did not fully address you concern in our last response. For the population-based ODE model, we did not vary the values of σ_i and γ_i across locations because there does not appear to be strong evidence that they vary across locations (even if they do vary with age, for instance). In the revised version we have cited these studies in support of this assumption: [1, 2, 3].
  However, to understand the impact of these parameters on model dynamics, we carried out additional experiment on the Facebook-County network in which these values vary by location. More specifically, we randomly draw each σ_i from a normal distribution with mean 0.4 and standard deviation 0.08, and we randomly draw each γ_i from a normal distribution with mean 0.2 and standard deviation 0.04. This allows both σ_i and γ_i to have reasonably large variations from location to location. We simulate epidemic dynamics using random initialization of the ODE model (i.e., initially 1% of all counties has 0.1% of its population infected), and we average the results over 50 trials. The final results we obtained are almost identical to the setting when σ_i and γ_i were constant. We attached two figures that demonstrate these results at the end of our response.

- *(Minor) pg. 28, line 719: the term "local graph clustering" is written as one word (missing spaces).*

Response: Thank you for pointing it out. This has been fixed.

References

[1] Lauer SA, Grantz KH, Bi Q, Jones FK, Zheng Q, Meredith H, et al. The Incubation Period of Coronavirus Disease 2019 (COVID-19) From Publicly Reported Confirmed Cases: Estimation and Application. Annals of internal medicine. 2020;172:577–582. doi:10.7326/M20-0504.

[2] Tan WYT, Wong LY, Leo YS, Toh MPHS. Does incubation period of COVID-19 vary with age? A study of epidemiologically linked cases in Singapore. Epidemiology and Infection. 2020;148:e197. doi:10.1017/S0950268820001995.

[3] Dhouib W, Maatoug J, Ayouni I, Zammit N, Ghammem R, Fredj SB, et al. The incubation period during the pandemic of COVID-19: a systematic review and meta-analysis. Systematic Reviews. 2021;10:101. doi:10.1186/s13643-021-01648-y.

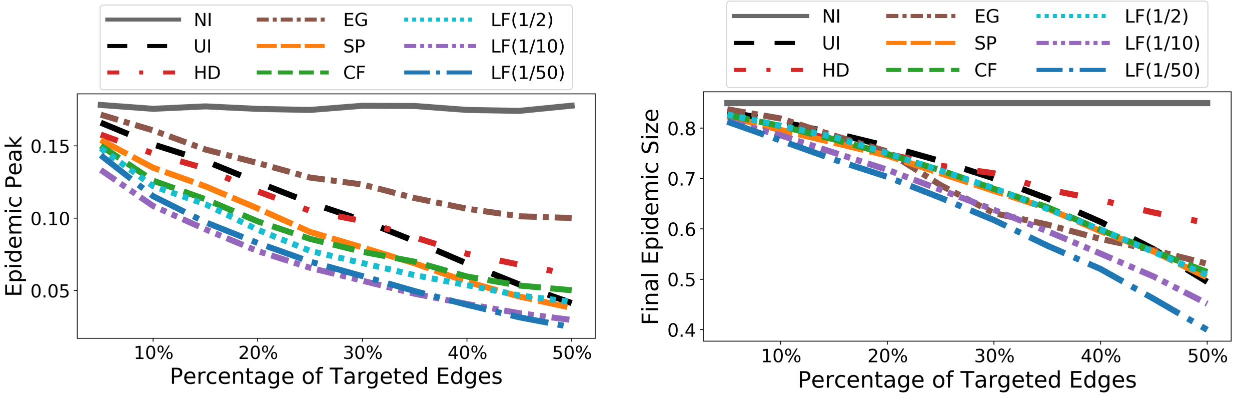

Supplement: Attachment — Submitted filename: Response Letter to Reviewers.pdf [file pcbi.1009351.s015.pdf]
